# SPINK6 inhibits human airway serine proteases and restricts influenza virus activation

**DOI:** 10.15252/emmm.202114485

**Published:** 2021-11-26

**Authors:** Dong Wang, Cun Li, Man Chun Chiu, Yifei Yu, Xiaojuan Liu, Xiaoyu Zhao, Jingjing Huang, Zhongshan Cheng, Shuofeng Yuan, Vincent Poon, Jian‐Piao Cai, Hin Chu, Jasper Fuk‐Woo Chan, Kelvin Kai‐Wang To, Kwok Yung Yuen, Jie Zhou

**Affiliations:** ^1^ Department of Microbiology Li Ka Shing Faculty of Medicine The University of Hong Kong Hong Kong China; ^2^ Applied Bioinformatics Center St Jude Children’s Research Hospital Memphis TN USA; ^3^ State Key Laboratory of Emerging Infectious Diseases The University of Hong Kong Hong Kong China; ^4^ Carol Yu Centre for Infection The University of Hong Kong Hong Kong China

**Keywords:** airway organoids, HA cleavage, influenza virus, SPINK6, virus maturation, Genetics, Gene Therapy & Genetic Disease, Microbiology, Virology & Host Pathogen Interaction

## Abstract

SPINK6 was identified in human skin as a cellular inhibitor of serine proteases of the KLK family. Airway serine proteases are required to cleave hemagglutinin (HA) of influenza A viruses (IAVs) to initiate an infection in the human airway. We hypothesized that SPINK6 may inhibit common airway serine proteases and restrict IAV activation. We demonstrate that SPINK6 specifically suppresses the proteolytic activity of HAT and KLK5, HAT‐ and KLK5‐mediated HA cleavage, and restricts virus maturation and replication. SPINK6 constrains the activation of progeny virions and impairs viral growth; and vice versa, blocking endogenous SPINK6 enhances HA cleavage and viral growth in physiological‐relevant human airway organoids where SPINK6 is intrinsically expressed. In IAV‐infected mice, SPINK6 significantly suppresses viral growth and improves mouse survival. Notably, individuals carrying the higher SPINK6 expression allele were protected from human H7N9 infection. Collectively, SPINK6 is a novel host inhibitor of serine proteases in the human airway and restricts IAV activation.

## Graphical abstract

Serine protease inhibitor Kazal‐type 6 (SPINK6) restricts influenza A virus (IAV) extracellular maturation via inhibiting proteolytic cleavage of the influenza virus hemagglutinin (HA) by human airway trypsin‐like protease (HAT) and kallikrein 5 (KLK5).

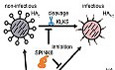

**The paper explained**

**Problem**

Respiratory protease/antiprotease balance determines susceptibility to viral infections, including influenza. SPINK6 was identified in human skin as a host inhibitor of serine proteases of the KLK family. We sought to address whether SPINK6 can suppress the proteolytic activation of influenza viruses in the human respiratory tract.

**Results**

SPINK6 specifically suppresses HAT and KLK5 activation of influenza viruses, and restricts virus maturation and replication. Blocking endogenous SPINK6 enhances HA cleavage and viral growth in physiologically relevant human airway organoids where SPINK6 is intrinsically expressed. In IAV‐infected mice, SPINK6 significantly suppresses viral growth and improves mouse survival.

**Impact**

SPINK6 is a novel host inhibitor of serine proteases in the human airway and restricts influenza virus activation. SPINK6 can be developed for the prevention and therapy of influenza infections.

## Introduction

Respiratory viral infections are major health threats globally. Seasonal influenza A viruses (IAVs), including pandemic 2009 A(H1N1) (H1N1/pdm), are the major cause of human respiratory infections, affecting 5–15% of the human population with ˜500,000 annual deaths worldwide (Novel Swine‐Origin Influenza *et al*, 2009). Human IAVs normally lead to upper respiratory infection with mild‐to‐moderate symptoms, and occasionally cause pneumonia with fatal outcomes. A novel avian A(H7N9) virus has caused recurrent outbreaks of human infection since 2013 (Chen *et al*, 2013). Human H7N9 infection commonly manifested as rapidly progressive pneumonia, with a case fatality rate higher than 30%.

Influenza virus infection is initiated by the surface glycoprotein hemagglutinin (HA) binding to cellular receptors, followed by the fusion of viral membrane and cellular endosomal membrane. HA is synthesized as a fusion‐inactive precursor protein HA0 that requires proteolytic cleavage by cellular proteases into disulfide‐linked HA1 and HA2 subunits. The exposed fusion domain in the N terminal of HA2 then undergoes membrane fusion (Klenk & Garten, 1994). HA0 of most human and avian influenza viruses contain a monobasic (a single arginine, or rarely a single lysine) cleavage site that is recognized by trypsin‐like serine proteases expressed abundantly in the human respiratory and gastrointestinal tract (Bottcher *et al*, 2006; Beaulieu *et al*, 2013); whereas highly pathogenic avian viruses including H5N1 contain a multi‐basic cleavage site that is cleaved by ubiquitous intracellular proteases such as Furin. Serine proteases are a predominant class among respiratory proteases. Type II transmembrane serine proteases HAT and TMPRSS2 are the major proteases responsible for HA cleavage in the human airway (Bottcher *et al*, 2006; Bottcher‐Friebertshauser *et al*, 2010, 2013). The role of these airway serine proteases for influenza virus replication has been extensively studied *in vitro* and *in vivo* (Tarnow *et al*, 2014).

A new family of serine proteases, the kallikrein (KLK)‐related peptidase family, comprises 15 secreted serine proteases (Prassas *et al*, 2015). Dysfunction of tissue‐specific regulation of KLK activity is linked to several pathologies, including skin barrier dysfunction, psychological disorder, pathological inflammation, and cancer (Fischer *et al*, 2014; Prassas *et al*, 2015; Zheng *et al*, 2017). Interestingly, Hamilton *et al* reported that KLK5 and KLK12 are secreted from the human respiratory tract, and can cleave the HA of H1, H2, and H3 subtypes (Hamilton & Whittaker, 2013). Magnen *et al* (2017) recently demonstrated that KLK5 promotes the infectivity of seasonal influenza virus H3N2 *in vitro* and *in vivo*. Collectively, both trypsin/chymotrypsin‐like serine proteases and some KLK serine proteases contribute to the activation and replication of influenza viruses in the human respiratory tract.

Meanwhile, there is a growing recognition that virus activation proteases are not the sole player during viral infection; respiratory protease/antiprotease balance determines susceptibility to viral infections, including influenza (Meyer & Jaspers, 2015). In contrast to an array of HA cleavage proteases mentioned above (Okumura *et al*, 2010; Baron *et al*, 2013), only a few antiproteases have been identified. Secretory leukocyte protease inhibitor (SLPI) is highly expressed by epithelial cells and immune cells and present in respiratory secretions; the inducible SLPI was protective during respiratory virus infections (Kido *et al*, 2004). Plasminogen activator inhibitor 1, encoded by Serpin E1 gene, inhibits HA cleavage and restricts maturation of progeny virions (Dittmann *et al*, 2015).

Serine protease inhibitor Kazal‐type 6 (SPINK6) was initially identified in human and mouse skin (Meyer‐Hoffert *et al*, 2010). It acts on most proteases of the KLK family, including KLK2, KLK5, KLK12, KLK13, and KLK14 (Kantyka *et al*, 2011). As a newly identified protease inhibitor, SPINK6 was reported to be involved in skin barrier function (Fischer *et al*, 2014) and metastasis of nasopharyngeal carcinoma (Zheng *et al*, 2017). Given its inhibition of multiple KLKs, more biological functions of SPINK6 are yet to be explored. As aforementioned, KLK5 and KLK12, the protease targets of SPINK6, can activate influenza viruses in the human respiratory tract (Hamilton & Whittaker, 2013). However, there is a gap in whether SPINK6 is indeed expressed in human respiratory cells, in which its inhibition of KLK5 and KLK12 is operational. In addition, SPINK6 inhibition of serine proteases may not be restricted to KLKs exclusively. It was documented that recombinant SPINK6 protein showed a moderate inhibitory effect on the proteolytic activity of bovine trypsin, and it suppressed the activity of trypsin‐like proteases in normal mouse keratinocytes (Lu *et al*, 2012; Fischer *et al*, 2014). The evidence, collectively, prompted us to elucidate whether SPINK6 is a novel inhibitor of common respiratory serine proteases and could suppress the activation of IAVs in the human airways.

## Results

### SPINK6 restricts proteolytic activity of trypsin, trypsin‐mediated HA cleavage, and viral replication

**Figure EV1:**
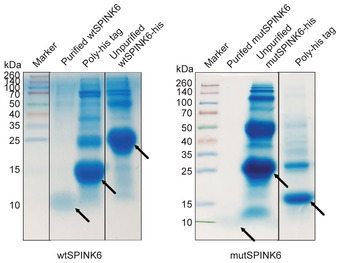
Coomassie blue staining shows the raw and purified recombinant proteins. Raw recombinant proteins of wtSPINK6 and mutSPINK6, purified wtSPINK6 and mutSPINK6 proteins, and removed poly‐His tag are indicated with arrows after SDS‐PAGE and Coomassie blue staining. Source data are available online for this figure.

**Figure 1:**
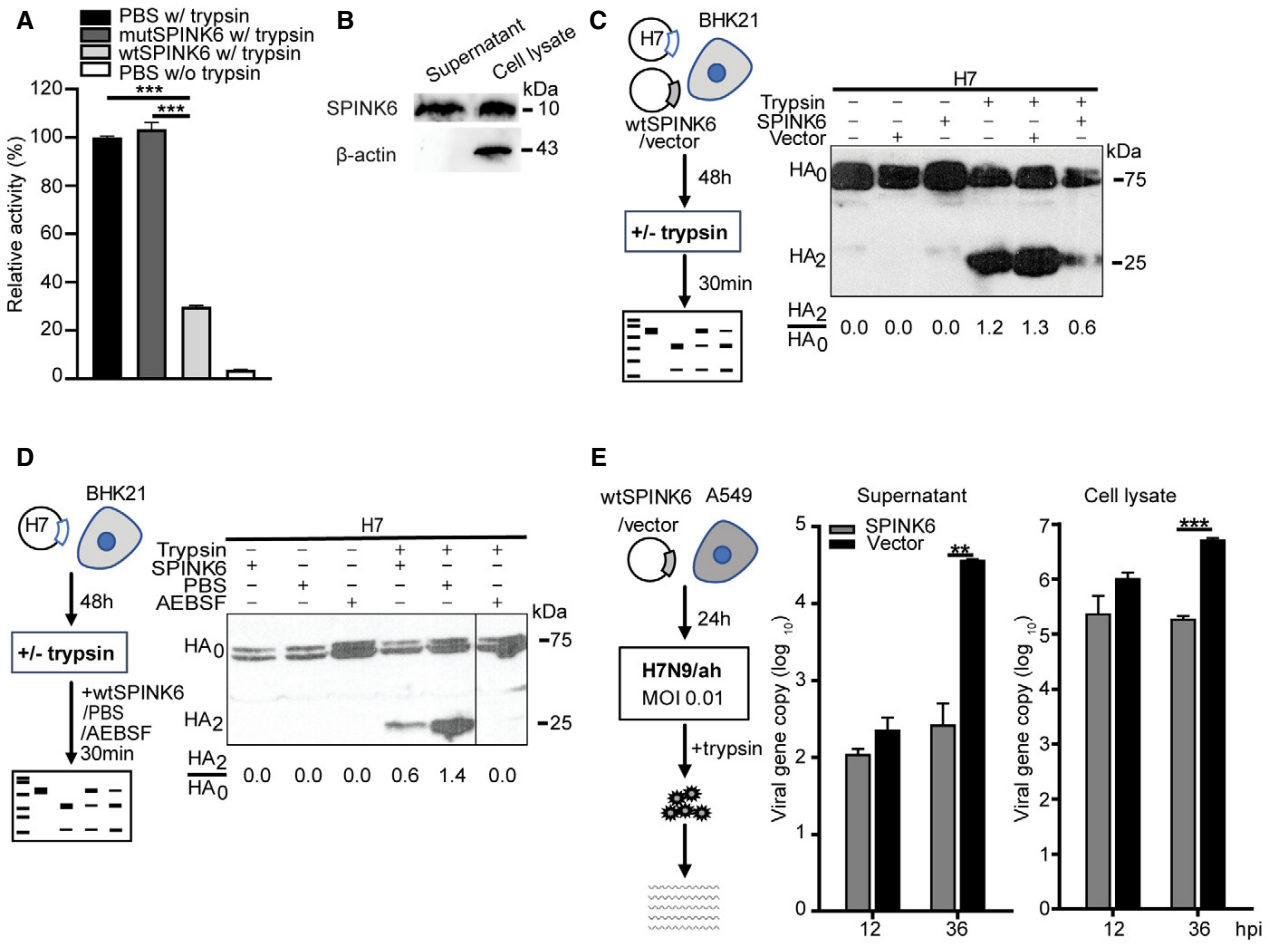
SPINK6 constrains proteolytic activity of trypsin, and trypsin‐mediated HA cleavage and viral growth. TPCK trypsin is premixed with wtSPINK6 protein or mutSPINK6 protein or PBS in triplicate, incubated with a fluorogenic substrate for 30 min, and then applied to fluorescence assay. Data are presented as mean and SD in a representative experiment performed three times. The fluorescence intensity of TPCK trypsin/PBS mixture is arbitrarily set as 1. ***P < 0.001.At 48 h post‐transfection of wtSPINK6 plasmid in 293T cells, cell lysate and concentrated supernatant (50×) were applied to the detection of SPINK6 protein and β‐actin by WB.At 48 h after co‐transfection of H7 plasmid with wtSPINK6 plasmid or blank vector, the transfectants were incubated with or without TPCK trypsin for 30 min, and then applied to examine HA cleavage by WB. Intensities of HA2 and HA0 bands are quantified with ImageJ. HA2/HA0 ratio of each sample is shown at the bottom.At 48 h after transfection of H7 plasmid, the transfectants were incubated with TPCK trypsin in the presence of recombinant wtSPINK6 protein or PBS or a protease inhibitor AEBSF for 30 min and then applied to examine HA cleavage.At 24 h after transfection of wtSPINK6 plasmid or blank vector in triplicate, A549 cells were inoculated with H7N9/ah virus. At the indicated hours post‐infection (hpi), culture media (supernatant) and cell lysate were harvested for viral load detection. Data are presented as mean and SD in a representative experiment performed three times. **P < 0.01; ***P < 0.001. Student’s *t*‐test. Source data are available online for this figure.

SPINK6 was initially identified to be a cellular inhibitor of serine proteases of the KLK family in human skin (Meyer‐Hoffert *et al*, 2010). We asked whether the inhibitory effect of SPINK6 on KLK5 and KLK12 is operational in other common respiratory serine proteases capable of HA activation. We performed a cell‐free serine protease activity assay using a fluorogenic substrate of serine proteases. TPCK trypsin, a prototype chymotrypsin‐like serine protease, is commonly used as a proxy for HA activation in cell culture‐based propagation of most IAVs. We tested whether SPINK6 can inhibit the proteolytic activity of TPCK trypsin. To this end, recombinant wild‐type SPINK6 (wtSPINK6) and mutant SPINK6 protein (mutSPINK6) were expressed in *E*. *coli* and purified (Fig EV1), the latter carrying loss‐of‐function mutations in the protease inhibition domain (Zheng *et al*, 2017). We found that, compared to mutSPINK6 and PBS, wtSPINK6 significantly reduced the proteolytic activity of TPCK trypsin (Fig 1A). SPINK6 is predicted to be a secreted protein. To clarify, we overexpressed SPINK6 in 293T cells, cell lysates and cell‐free medium were harvested for Western blot analysis. As shown in Fig 1B, SPINK6 is indeed a secreted protein.

As aforementioned, proteolytic cleavage of HA is a prerequisite for IAV to initiate a productive infection in host cells. We then evaluated the effect of SPINK6 on TPCK trypsin‐mediated HA cleavage. To this end, a plasmid encoding the HA of H7N9/ah virus (H7) was co‐transfected with SPINK6 plasmid or a blank vector into BHK21 cells, which are devoid of endogenous proteases able to cleave HA. As a low pathogenic strain in domestic poultry, H7N9/ah virus possesses a monobasic cleavage site (Gao *et al*, 2013). At 48 h post‐transfection, the transfected cells treated or mock treated with TPCK trypsin were harvested for detection of HA cleavage by Western blot. As shown in Fig 1C, in the absence of TPCK trypsin, H7 presented as an uncleaved HA0, no matter SPINK6 plasmid or the vector was co‐transfected. Upon TPCK trypsin treatment, in both mock‐transfected cells and vector‐transfected cells, H7 was readily cleaved, with a HA2/HA0 ratio of 1.2 and 1.3, respectively. Interestingly, H7 cleavage by TPCK trypsin was restricted upon SPINK6 overexpression, with a notably reduced HA2/HA0 ratio of 0.6. Moreover, the addition of recombinant wtSPINK6 protein remarkably constrained trypsin‐mediated H7 cleavage in comparison to PBS, with a HA2/HA0 ratio of 0.6 versus 1.4 (Fig 1D). AEBSF, a commercial inhibitor of serine proteases, was used as a positive control for inhibiting TPCK trypsin‐mediated HA cleavage. We also inspected the multiple‐cycle replication of H7N9/ah sustained by TPCK trypsin after overexpressing SPINK6 or the blank vector (Fig 1E). We observed an active replication of H7N9/ah upon transfection of the blank vector. In contrast, in SPINK6 overexpression cells, viral loads in culture media (supernatant) and cell lysate barely increased over time, and were significantly lower than those in vector‐transfected cells. Collectively, SPINK6 overexpression significantly abolished trypsin‐mediated HA cleavage and viral growth.

### SPINK6 suppresses proteolytic activities of HAT and KLK5, and constrains HAT‐/KLK5‐mediated HA cleavage

**Figure 2:**
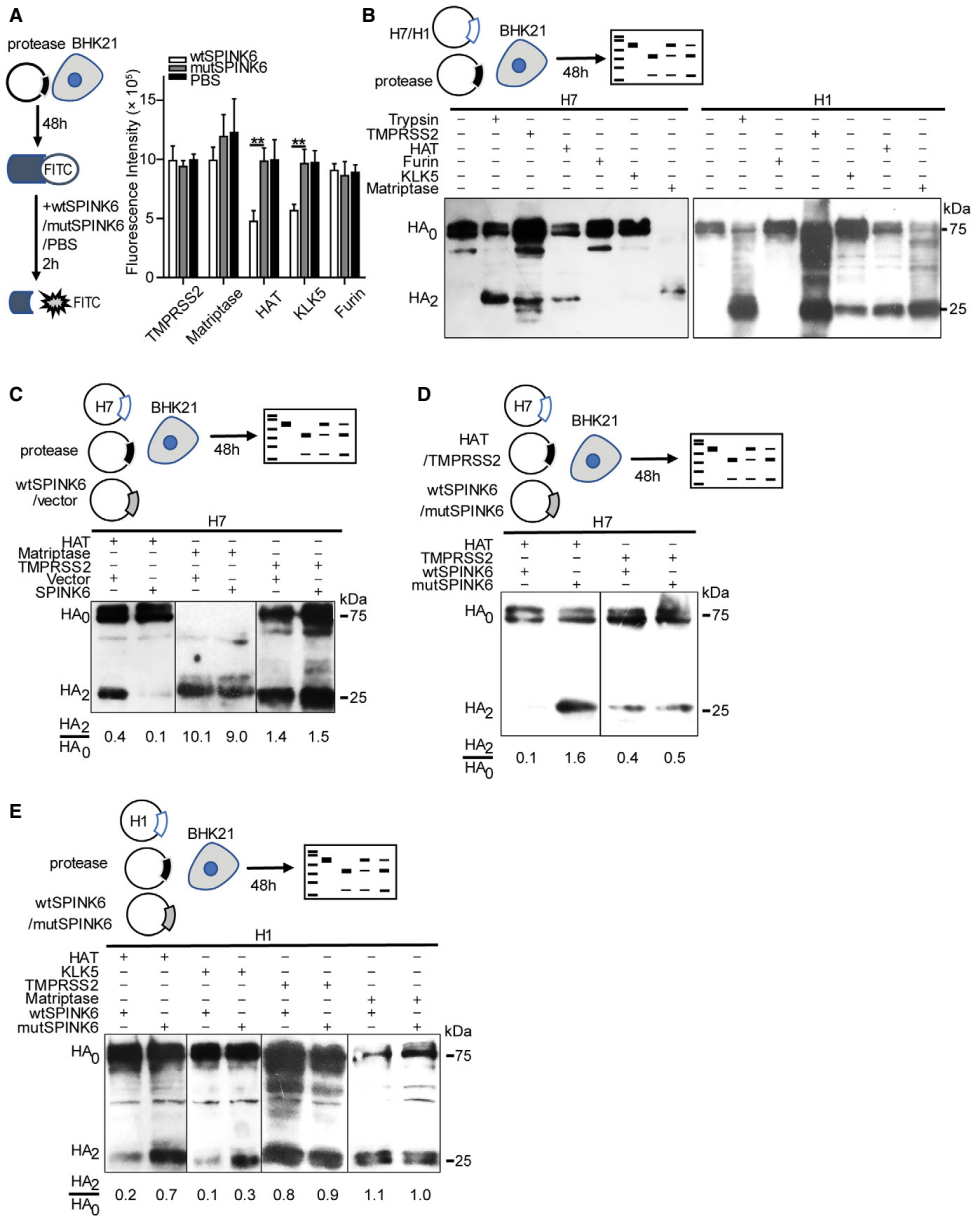
SPINK6 inhibits HAT and KLK5, and HAT‐ and KLK5‐mediated HA cleavage. At 48 h post‐transfection of the indicated protease plasmids in triplicate, BHK21 cells were incubated with a fluorogenic substrate and wtSPINK6 or loss‐of‐function mutSPINK6 or PBS for 2 h and then applied to fluorescence assay. Data represent mean and SD of triplicated wells in a representative experiment performed three times. **P < 0.01. Student’s *t*‐test.At 48 h after co‐transfection of the indicated protease plasmids and H7 or H1 plasmid, BHK21 cells were lysed to examine HA cleavage with WB.At 48 h after triple transfection of H7, the indicated proteases, and wtSPINK6 or vector, BHK21 cells were lysed for the detection of HA cleavage.At 48 h after triple transfection of H7, HAT or TMPRSS2, and wtSPINK6 or mutSPINK6, BHK21 cells were lysed for the detection of HA cleavage.At 48 h after triple transfection of H1, the indicated proteases, and wtSPINK6 or mutSPINK6, BHK21 cells were lysed for the detection of HA cleavage. Source data are available online for this figure.

To identify the target proteases of SPINK6, we overexpressed common HA activation serine proteases in BHK21 cells. At 48 h post‐transfection, the cells were incubated with the fluorogenic substrate for 2 h in the presence of recombinant wtSPINK6 or mutSPINK6 protein or PBS (Fig 2A). Compared to mutSPINK6 and PBS, wtSPINK6 significantly reduced the activities of HAT and KLK5; whereas it showed a negligible effect on TMPRSS2, Matriptase, and Furin. Thus, SPINK6 specifically and significantly inhibits proteolytic activities of serine protease HAT and KLK5. We then assessed whether SPINK6 inhibition of the proteolytic activity of serine proteases could lead to a compromised HA cleavage. To this end, we first tested which serine proteases can activate HA of H7N9/ah and H1N1/pdm. As shown in Fig 2B, H7 is cleaved after overexpression of TMPRSS2, HAT, and Matriptase, while Furin and KLK5 are unable to cleave H7. Besides HAT, TMPRSS2, and Matriptase, KLK5 can also cleave H1, which is consistent with a previous report (Hamilton & Whittaker, 2013). Subsequently, co‐transfection of three plasmids, including H7, proteases, and SPINK6 or vector, was performed to evaluate the effect of SPINK6 on HA cleavage by these proteases (Fig 2C). Compared to the blank vector, wtSPINK6 overexpression largely abolished HAT‐mediated H7 cleavage (HA2/HA0 ratio of 0.4 vs. 0.1), whereas H7 cleavage by TMPRSS2 or Matriptase was similar no matter wtSPINK6 or vector was overexpressed. We verified the result using mutSPINK6 plasmid to replace the blank vector. wtSPINK6, but not mutSPINK6, specifically restricted H7 cleavage by HAT. In contrast, wtSPINK6 and mutSPINK6 showed a similar effect on H7 cleavage by TMPRSS2 (Fig 2D).

SPINK6‐specific inhibition of HA cleavage by HAT, but not TMPRSS2, was reproduced in the cleavage of H1 (Fig 2E). Compared to mutSPINK6 overexpression, wtSPINK6 overexpression largely constrained H1 cleavage by HAT. Interestingly, H1 cleavage by KLK5 was notably attenuated upon wtSPINK6 overexpression. Likewise, wtSPINK6 overexpression had a minimal effect on TMPRSS2‐ and Matriptase‐mediated H1 cleavage. Thus, the role of SPINK6 on HA cleavage by the serine proteases exactly recapitulated its inhibition of proteolytic activities of these proteases as shown in Fig 2A.

### SPINK6 attenuates IAV replication by restricting virus maturation

**Figure 3:**
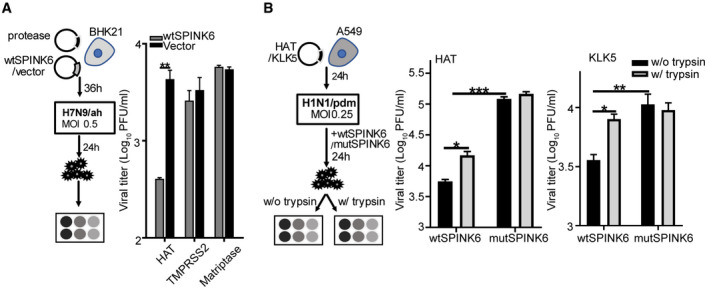
SPINK6 inhibits virus growth and maturation‐mediated HAT and KLK5. At 36 h after transfection of the indicted protease and wtSPINK6 plasmids or blank vector in triplicate, BHK21 cells were inoculated with H7N9/ah at a MOI of 0.5. Cell‐free culture media were harvested at 24 hpi for viral titration. Data represent the mean and SD of the triplicated wells in a representative experiment performed three times. **P < 0.01. Student’s *t*‐test.At 24 h after transfection of HAT or KLK5 plasmid in sextuplicate, A549 cells were inoculated with H1N1/pdm at MOI of 0.25. The infected cells were incubated with recombinant wtSPINK6 or mutSPINK6 protein in triplicate for 24 h. Culture media in each well were stored in two aliquots, one was applied to viral titration with conventional plaque assay (black bars) and the other was pre‐treated with TPCK trypsin for 1 h prior to plaque assay (grey bars). Data represent the mean and SD of the triplicated wells in a representative experiment performed three times. *P < 0.05; **P < 0.01; ***P < 0.001. Student’s *t*‐test.

To assess the effect of SPINK6 on protease‐driven IAV replication, we inoculated BHK21 cells with H7N9/ah at 36 h after co‐transfection of HAT, or TMPRSS2 or Matriptase, together with SPINK6 or vector. Cell‐free culture media were harvested at 24 h post‐infection for detecting viral growth (Fig 3A). SPINK6 overexpression significantly reduced HAT‐mediated viral growth, whereas it exerted a minimal effect on viral replication sustained by TMPRSS2 and Matriptase. We further verified SPINK6 inhibition of protease‐driven viral growth in more susceptible A549 cells upon infection with H1N1/pdm. IAVs with a monobasic HA cleavage site, including H1N1/pdm, barely replicate in A549 cells in the absence of exogenous proteases such as TPCK trypsin. At 24 h after transfection of HAT or KLK5, A549 cells inoculated with H1N1/pdm were incubated in the presence of wtSPINK6 or mutSPINK6 for 24 h. The culture medium in each well was harvested and stored in two aliquots; one aliquot was applied to the conventional plaque assay for viral titration (Fig 3B). wtSPINK6 treatment in HAT overexpression cells resulted in a significantly decreased viral titer of more than 1 log_10_ unit than mutSPINK6 (HAT, black bars in wtSPINK6 vs. mutSPINK6). A significant viral reduction resulted from wtSPINK6 treatment was reproduced in KLK5‐driven viral growth (KLK5, black bars in wtSPINK6 vs. mutSPINK6).

The other aliquots of the same medium samples were simultaneously applied to a modified plaque assay in which the media were treated with TPCK trypsin prior to inoculation onto MDCK monolayers. Notably, TPCK trypsin pretreatment significantly rescued wtSPINK6‐induced viral reduction in both HAT and KLK5 overexpression cells (black vs. grey bars in wtSPINK6 treatment), suggesting that the compromised viral growth in wtSPINK6‐treated cells was indeed attributed to the accumulation of non‐infectious progeny virions with uncleaved HA0. In stark contrast, trypsin pretreatment had a minimal effect on the infectivity of progeny virions released from mutSPINK6‐treated cells (black vs. grey bars in mutSPINK6 treatment). Collectively, SPINK6 suppressed viral growth by restricting virus maturation.

### SPINK6 suppression of virus activation and growth occurs in human airway organoids

**Figure EV2:**
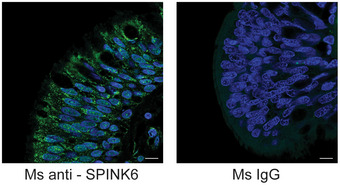
SPINK6 is expressed in the airway epithelial cells in human bronchial tissue. Paraffin slides of human lung tissues are stained with an α‐SPINK6 (green, left) or an isotopic IgG (right) and applied to confocal imaging. Nuclei are counterstained with DAPI (blue). Scale bar, 10 µm.

**Figure 4:**
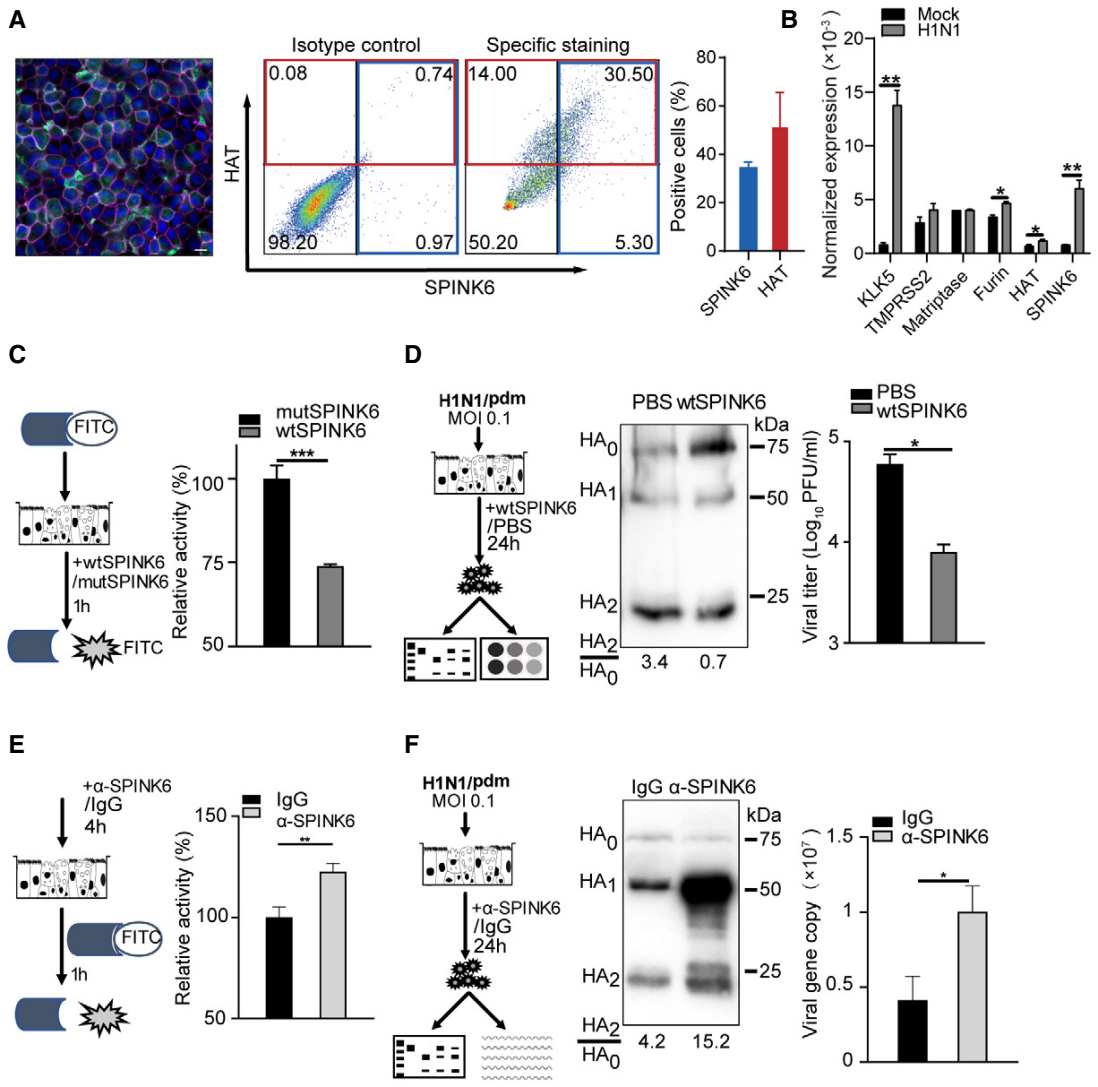
Exogenous and endogenous SPINK6 affect virus maturation and viral replication in human airway organoids. Confocal imaging (left) shows intrinsic SPINK6 (green) expression in the epithelial cells in 2D human airway organoids. Nuclei and actin filaments are counterstained and shown as blue and purple, respectively. Scale bar, 10 µm. Flow cytometry analysis demonstrates HAT and SPINK6 expression in human airway organoids. The plots (middle) show the representative result of one experiment performed three times. The percentages of HAT and SPINK6 positive cells (right) are shown as mean and SD of three independent experiments.At 24 h after inoculation of H1N1/pdm or mock inoculation in triplicate, 2D human airway organoids were harvested to detect mRNA expression levels of the indicated genes normalized with GAPDH. Data represent the mean and SD of a representative experiment performed three times.2D human airway organoids were incubated with the fluorogenic substrate in triplicate for 1 h in the presence of wtSPINK6 or mutSPINK6, and applied to fluorescence assay. The fluorescence intensity of mutSPINK6‐treated organoids is arbitrarily set as 1. Data represent the mean and SD of a representative experiment performed three times.After inoculation of H1N1/pdm, 2D human airway organoids were incubated with wtSPINK6 or PBS in triplicate for 24 h (left). Cell‐free culture media were harvested for viral titration (right) and examination of HA cleavage by WB after 10‐fold concentration (middle). Viral titer data present the mean and SD of triplicated samples.2D airway organoids were applied to fluorescence assay after incubation with α‐SPINK6 or isotypic IgG in triplicate for 4 h (left). Data present the mean and SD of triplicated samples.After inoculation of H1N1/pdm, 2D human airway organoids were incubated with an α‐SPINK6 or isotype IgG in triplicate for 24 h (left). Cell‐free culture media were harvested for viral load detection (right) and examination of HA cleavage by WB after 10‐fold concentration (middle). Viral load data present the mean and SD of triplicated samples. Data information: *P < 0.05; **P < 0.01; ***P < 0.001. Student’s *t* test. Source data are available online for this figure.

We performed immunofluorescence staining and found that SPINK6 is indeed expressed in the airway epithelial cells in human lung tissues (Fig EV2). We have reported the establishment of human airway organoids from adult stem cells in primary lung tissues. The non‐differentiated airway organoids can be consecutively and stably expanded *in vitro* for over 1 year; upon induction of maturation, the differentiated airway organoids can morphologically and functionally simulate human airway epithelium to a near physiological level. Furthermore, the differentiated airway organoids sustain robust replication of IAVs in the absence of TPCK trypsin since they possess endogenous HA activation serine proteases (Zhou *et al*, 2018). To understand the role of SPINK6 in the course of IAV infection, we first inspected the expression profile of SPINK6 in the differentiated airway organoids. Immunofluorescence staining clearly reveals SPINK6 expression in the epithelial cells in differentiated 2D airway organoids (Fig 4A). Flow cytometry analysis shows that over 30% of cells in the differentiated airway organoids express SPINK6, and a large proportion of SPINK6‐positive cells co‐express HAT. We also examined the expression profile of HA activation proteases and SPINK6 upon H1N1/pdm infection in airway organoids. We observed a significant upregulation of HAT and Furin, especially KLK5, after infection, whereas TMPRSS2 and Matriptase were barely stimulated (Fig 4B). A significant elevation of SPINK6 transcripts was observed in airway organoids upon H1N1/pdm infection.

We next evaluated whether SPINK6 could inhibit the proteolytic activity of endogenous serine proteases in differentiated airway organoids. To this end, 2D airway organoids were incubated with the fluorogenic substrate in the presence of wtSPINK6 or mutSPINK6 protein for 1 h, and then applied to the serine protease activity assay (Fig 4C). Compared to mutSPINK6, wtSPINK6 significantly suppressed the proteolytic activity of endogenous serine proteases in airway organoids. We then asked whether the compromised protease activity in these organoids had any sequential effect on HA cleavage and viral growth. Thus, H1N1/pdm‐infected airway organoids were incubated with wtSPINK6 protein or PBS for 24 h. Cell‐free culture media containing progeny virions were harvested for viral titration and examination of HA cleavage by Western blot using a mouse antiserum against H1N1/pdm (Fig 4D). We observed a dramatically reduced HA2/HA0 ratio upon wtSPINK6 treatment versus PBS treatment (0.7 vs. 3.4), indicating that more progeny virions of non‐infectious with uncleaved HA0 were produced from the wtSPINK6‐treated organoids than those treated with PBS. Thus, SPINK6 treatment indeed impaired HA cleavage in authentic virus particles. Consistently, SPINK6 treatment resulted in a significantly reduced viral titer after multi‐cycle replication.

As demonstrated above, SPINK6 is intrinsically expressed in bronchial epithelial cells in human lung tissue and human airway organoids. To assess the role of endogenous SPINK6, we treated the organoids with an antibody that can block the protease inhibition domain of SPINK6, and then examined serine protease activity, HA cleavage, and replication kinetics of H1N1/pdm. The addition of the α‐SPINK6 onto 2D airway organoids significantly increased the proteolytic activity compared to the isotype IgG (Fig 4E). Accordingly, blocking endogenous SPINK6 activity substantially enhanced HA cleavage in progeny virions produced from the airway organoids, as shown by a dramatically increased HA2/HA0 ratio of 15.2 versus 3.4. Likewise, the addition of the α‐SPINK6 significantly potentiated viral growth (Fig 4F).

### SPINK6 treatment suppresses viral growth and improves survival in IAV‐infected mice

**Figure 5:**
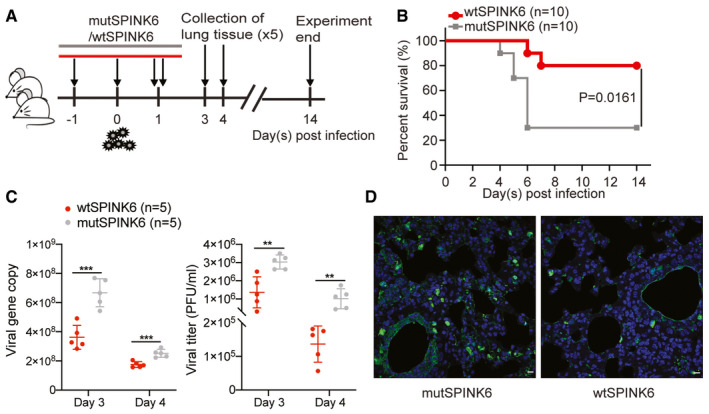
SPINK6 treatment suppresses viral growth and improves survival in mouse influenza infection. Regime of the mouse experiment. Balb/c mice were a mouse‐adapted strain of pandemic H1N1 virus. At 24 h before inoculation, and 8, 24, and 36 h post‐inoculation, two groups of mice were intranasally administered with wtSPINK6 protein or mutSPINK6 protein. Ten mice treated with wtSPINK6 or mutSPINK6 were monitored daily for disease signs, body weight, and survival for 14 days. Five mice treated with wtSPINK6 or mutSPINK6 were sacrificed at 3 and 4 days after the viral challenge. Lung tissues were collected for the quantification of viral growth and immunofluorescence staining.Survival rates of mice treated with wtSPINK6 and mutSPINK6 were analyzed with Mantel–Cox test.Viral load and viral titer in the lung homogenates of mice treated with wtSPINK6 and mutSPINK6. Data represent mean ± SD. **P < 0.01; ***P < 0.001. Student’s *t‐*test.Mouse lung tissues are applied to immunofluorescence staining to identify the viral NP‐positive cells (green). Nuclei are counterstained with DAPI (blue). Representative confocal images of virus‐infected cells in the indicated mice. Scale bar, 10 µm.

**Figure EV3:**
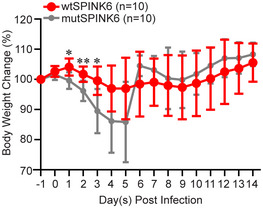
SPINK6 treatment alleviated body weight loss in IAV‐infected mice. After IAV inoculation, body weights of 10 mice treated with wtSPINK6 or mutSPINK6 were monitored. Daily body weigh changes in survived mice are presented. Data represent mean ± SD. *P < 0.05; **P < 0.01. Student’s *t*‐test is used for data analysis.

To verify SPINK6 inhibition of virus activation and growth *in vivo*, we treated two groups of Balb/c mice with recombinant wtSPINK6 or mutSPINK6 via intranasal administration and monitored disease sign, viral growth, and mouse survival after challenging with a mouse‐adapted strain of H1N1/pdm virus (Fig 5A). Considering the respiratory tract to be the primary infection site, we performed pilot experiments. We elected a scheme in which 10 µg recombinant SPINK6 proteins in a volume of 20 µl were administered intranasally 4 times prior to and after virus inoculation (Fig 5A). We found that the virus‐infected mice treated with mutSPINK6 developed an enhanced severity of illness than those treated with wtSPINK6, such as hunching, labored breathing, and fur ruffling; the former also experienced more body weight loss than the latter (Fig EV3). Notably, only 30% of mice treated with mutSPINK6 survived the infection; while the survival rate was 80% in wtSPINK6‐treated mice (Fig 5B). On day 3 and day 4 post‐infection, the viral loads and titers in lung tissues were significantly higher in mutSPINK6‐treated mice than those treated with wtSPINK6 (Fig 5C). Furthermore, immunofluorescence staining revealed more virus‐infected cells in lung tissues of the former than the latter (Fig 5D).

### A genetic variant correlated to higher SPINK6 expression is significantly associated with protection from human H7N9 infection

We previously conducted a small‐scale genome‐wide association study (GWAS) in H7N9 patients and controls of healthy poultry workers who were heavily exposed to the virus during the early outbreak in 2013 to identify the genetic variations predisposing to human H7N9 influenza (Chen *et al*, 2015). GWAS results revealed that an intergenic single nucleotide polymorphism (SNP) upstream of *SPINK6*, rs1432689 was significantly associated with the susceptibility to H7N9 infection (Table 1). The allele C is significantly associated with a higher susceptibility to the infection; the individuals carrying C/C and C/T genotype show a higher risk of 1.78‐fold to H7N9 infection than those carrying T/T genotype.

**Table 1:** Genetic variation of SPINK6 SNP rs1432689 is associated with the susceptibility to human H7N9 infection.

Variant distribution & analysis	H7N9 patients (*n* = 102)	Healthy controls (*n* = 106)
Genotype distribution, *n* (%)		
CC	61 (59.8)	44 (41.5)
CT	33 (32.4)	48 (45.3)
TT	8 (7.8)	14 (13.2)
Allelic analysis (allele C)		
OR	1.44	
*P* value	0.00852	
Genotype analysis (CC+CT)		
OR	1.78	
*P* value	0.00834	

OR, odds ratio.

**Figure 6:**
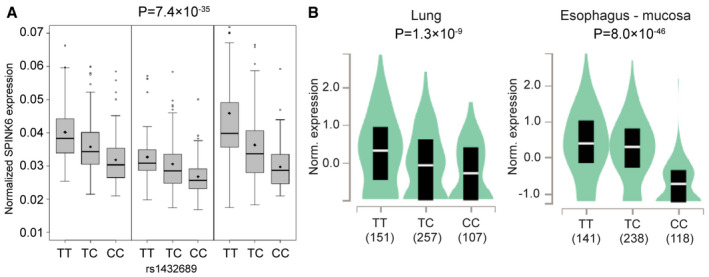
The association SNP genotypes are correlated with differential SPINK6 mRNA expression in human tissues. The association SNP rs1432689 genotypes are correlated with differential SPINK6 mRNA expression in over 1,000 human lung tissues from three centers. The box denotes interquartile range; the thick line and diamond within the box are the median and mean, respectively; whiskers are the minimum and maximum and open dots are outliers. Linear regression was used for eQTL data analysis. Data of three cohorts were applied to meta‐analysis and resulted in the P‐value of the association SNP.rs1432689 genotypes are correlated with SPINK6 expression in human lung tissues and esophagus mucosa from GTEx database. Sample sizes for each genotype are shown in the x‐axis. The y‐axis shows the normalized expression of SPINK6 (Norm. expression) in the indicated trusses. Median and interquartile range are represented by a white line and dark box, respectively. Data distribution is colored with light blue; a wider section of the violin plot indicates the data on the section have a higher frequency. Linear regression was used for data analysis.

Interestingly, the association SNP rs1432689 is a strong expression quantitative trait locus (eQTL) in the human lung tissues of more than 1,000 patients from three collaborative centers (Hao *et al*, 2012). Specifically, carriers of protective T allele show higher SPINK6 expression than those carrying risk C allele (Fig 6A). eQTL datasets, which have been established for various human primary cells and tissues (Cookson *et al*, 2009; Consortium GT, 2020), reveal the genome‐wide correlation of genetic variants with expression levels of host genes. In GTEx portal, a comprehensive public resource of tissue‐specific gene expression (https://www.gtexportal.org/home/snp/rs1432689), a strong correlation of rs1432689 genotypes with differential SPINK6 mRNA expression levels in human lung tissue and esophagus mucosa is revealed as well (Fig 6B). Moreover, based on HaploReg annotation (Ward & Kellis, 2016), rs1432689 and many variants in high linkage disequilibrium are located in the enhancer and promoter region of SPINK6, indicating that rs1432689 tags a regulatory haplotype dictating SPINK6 expression. Thus, the integration of the GWAS result and eQTL datasets suggested that higher SPINK6 expression derived from genetic variations may confer protection from human H7N9 infection.

## Discussion

SPINK6 was initially identified in the human skin as a cellular protein that inhibits serine proteases of the KLK family (Meyer‐Hoffert *et al*, 2010). SPINK6 was linked to pathologies, including skin barrier function (Fischer *et al*, 2014) and metastasis of nasopharyngeal carcinoma (Zheng *et al*, 2017). Interestingly, Fischer *et al* (2014) reported that SPINK6 inhibited proteolytic activities of trypsin‐like proteases in mouse keratinocytes. Using a cell‐free serine protease activity assay, we demonstrate that SPINK6 indeed inhibits proteolytic activity of TPCK trypsin, a prototype chymotrypsin‐like serine protease (Fig 1A). Consistently, TPCK trypsin‐mediated H7 cleavage and H7N9 replication are remarkably attenuated upon SPINK6 overexpression or the addition of recombination SPINK6 protein (Fig 1C–E). Apart from the known protease target KLK5, SPINK6 specifically inhibits the proteolytic activity of HAT (Fig 2A). In addition, SPINK6 overexpression largely abolishes H7 cleavage by HAT (Fig 2C and D). SPINK6‐specific inhibition of HAT and KLK5 was recapitulated in HA cleavage of H1N1/pdm virus (Fig 2E). Moreover, SPINK6 overexpression significantly attenuates HAT‐driven H7N9 replication (Fig 3A), and treatment with recombinant SPINK6 protein profoundly restricts H1N1/pdm replication driven by HAT and KLK5 (Fig 3B). Of note, the substantial viral reduction is attributed to the findings that SPINK6 treatment significantly impairs virus maturation, generating more non‐infectious progeny virions (Figs 3B and 4D).

We are prompted to address an intriguing question. Why does SPINK6 specifically inhibit HAT (also called TMPRSS11D), but not TMPRSS2? Both are type II transmembrane serine proteases predominantly distributed in the human respiratory and intestinal tract. Moreover, HAT, TMPRSS2, and other trypsin‐like serine proteases share a highly conserved catalytic protease domain (Bugge *et al*, 2009). We believe the distinct site of action of HAT and TMPRSS2 results in the difference. HAT cleaves newly synthesized HA before or during virus release, and HA of incoming virions prior to endocytosis on the cell surface, whereas TMPRSS2 cleaves newly synthesized HA within cells, but barely activates HA of incoming virions. Accordingly, inhibition of TMPRSS2 requires cellular uptake of synthetic protease inhibitors (Garten *et al*, 2015). We and others demonstrate SPINK6 is a secreted protein (Fig 1B; Zheng *et al*, 2017). Thus, SPINK6 exerts its inhibitory effect on HAT that is mainly functional on the cell surface, rather than TMPRSS2 operational in the intracellular membrane.

Of note, SPINK6 is intrinsically expressed in the bronchial epithelial cells in human lung tissues (Fig EV2) and human airway organoids (Fig 4A), a novel *in vitro* model of human respiratory epithelium established by us. Airway organoids provide a unique and physiological‐active model in which the biology of native human respiratory epithelial cells in the context of microbial invasion could be dissected readily. KLK5 and HAT are significantly upregulated in airway organoids upon H1N1/pdm infection, suggesting the importance of these proteases in the human airway during influenza infection (Fig 4B). SPINK6 inhibition of HA cleavage and viral replication was adequately reproduced in human airway organoids. The addition of recombinant SPINK6 protein significantly diminishes serine protease activity of the organoids, restricts HA cleavage in authentic progeny virions produced from the infected organoids, and attenuates viral growth (Fig 4C and D). More importantly, blocking endogenous SPINK6 activity with an antibody promotes HA cleavage and viral growth, highlighting the role of endogenous SPINK6 in the human airway during IAV infection (Fig 4E and F). SPINK6‐mediated virus inhibition was verified in mice challenged with the mouse‐adapted H1N1/pdm virus. Treatment of wtSPINK6 significantly reduces viral growth and improves mouse survival (Fig 5). Notably, an integrative analysis of the GWAS result and eQTL datasets suggested that higher SPINK6 expression conferred significant protection from human H7N9 infection (Table 1 and Fig 6).

Although many serine proteases have been demonstrated to mediate HA activation *in vitro*, HAT and TMPRSS2 are the major serine proteases in the human respiratory epithelium, and thus play a crucial role in IAV infection (Meyer & Jaspers, 2015). SPINK6‐specific inhibition of HAT, and its native target KLK5 and KLK12, as well as its upregulation upon infection, underscores the importance of SPINK6 in human influenza infection. As aforementioned, respiratory protease/antiprotease balance determines susceptibility to viral infections, including influenza. SLPI is believed to provide a significant component of the human antiprotease shield within the lung (Kido *et al*, 2004). However, HAT is poorly inhibited by SLPI (Yasuoka *et al*, 1997). In this regard, SPINK6 inhibition of HAT and KLKs may represent a critical host defense mechanism to restrict IAV activation and replication in the human airway. Taken together, we identify SPINK6 to be a novel cellular inhibitor of serine proteases that can restrict the activation and propagation of IAVs in the human respiratory tract.

## Materials and Methods

### Cell lines, viruses, plasmids, and antibodies

BHK21, 293T, and A549 cells are purchased from ATCC and maintained in Dulbecco's modified Eagle's medium (DMEM) (Gibco) supplemented with 10% fetal bovine serum (FBS) (Gibco), 100 units/ml penicillin, and streptomycin at 37°C with 5% CO_2_. Influenza A virus A/Anhui/1/2013 (H7N9) (H7N9/Ah) and A/Hong Kong/415742/2009 (H1N1) (H1N1/pdm) were used for *in vitro* experiments; a mouse‐adapted strain A/Hong Kong/415742Md/2009 (H1N1) virus was used in mouse experiments. The viruses were propagated in embryonated eggs, titrated, and stored in −80°C. The ORF cDNAs of human wild‐type SPINK6, mutant SPINK6 with a loss‐of‐function R19A mutation, HAT, KLK5, Matriptase, and Furin were inserted into a pCMV6 vector. Flag‐tagged human TMPRSS2 ORF cDNA expression plasmid, His‐tagged codon‐optimized HA mammalian expression plasmids of A/California/07/2009(H1N1) and A/Anhui/1/2013(H7N9) virus, and polyclonal antibodies against these HAs, α‐H_1_ (A/California/04/2009, 11055‐T62) and α‐H_7_ (A/Anhui/1/2013, 40103‐RP02) were purchased from Sino Biological. All experiments with live viruses were conducted in biosafety level 2 or 3 laboratories upon institutional approval.

### Expression, preparation, and purification of recombination proteins

293T cells in a six‐well plate were transfected with 5 µg wtSPINK6 plasmid in DMEM medium supplemented with 10%FBS. At 48 h after transfection, 50‐fold concentrated culture medium and whole‐cell lysates were harvested in RIPA lysis buffer for Western blot analysis using an α‐SPINK6 (Abnova, H00404203‐M04) as we described previously (Zhou *et al*, 2019). The ectodomain of wtSPINK6 and mutSPINK6 with a His affinity tag was cloned into a pET32a vector and then propagated in E. coli BL21 (DE3) cells to express recombinant wtSPINK6 and mutSPINK6 proteins. His‐tagged proteins were obtained after purification using Ni‐NTA agarose resin column (Qiagen). After concentration with Centrifugal Filter Unit (UFC901024, 10 KDa, Amicon), the eluents were incubated with TEV protease (T4455‐10KU, Sigma Aldrich) overnight at 4°C to remove the His‐tag. The purified proteins were verified by SDS‐PAGE and Coomassie blue staining.

### Serine protease activity assay

In the cell‐free protease activity assay, a volume of 10 µl TPCK trypsin (50 ng/µl) premixed with 500 ng/µl wtSPINK6 or mutSPINK6 or PBS in triplicate was dispersed into a well of 96‐well plate preloaded with 100 µl 2.5 µM Peptidyl‐MCA substrate (Boc‐Leu‐Gly‐Arg‐AMC, BACHEM). After incubation at 37°C for 30 min, the solution was applied to fluorescence assay in a Victor X3 Multilabel reader (PerkinElmer). To identify the protease targets of SPINK6, we seeded BHK21 cells in a 96‐well plate and transfected them with 100 ng TMPRSS2 or HAT plasmid, or 50 ng Matriptase plasmid, or 250 ng KLK5 plasmid per well. At 48 h post‐transfection, the cells were incubated for 2 h in fresh DMEM medium supplemented with 2.5 µM Peptidyl‐MCA substrate and 5 µg wtSPINK6 or mutSPINK6 protein or PBS in triplicated wells, and followed with fluorescence assay. 2D airway organoids were incubated with culture media supplemented with 2.5 µM Peptidyl‐MCA substrate and 5 µg wtSPINK6 or mutSPINK6 protein for 1 h, and then applied to fluorescence assay to assess the effect of SPINK6 on protease activity. Alternatively, 2D human airway organoids were pretreated with 20 µg/ml α‐SPINK6 (Abcam, ab201319) or isotype rabbit IgG (Abcam, ab37415) in triplicate for 4 h, followed by 1 h incubation with 2.5 µM Peptidyl‐MCA substrate, and then applied to fluorescence assay.

### Examination of HA cleavage

We assessed the effect of SPINK6 on trypsin‐mediated H7 cleavage. At 4 h after co‐transfection of 1 µg H7 plasmid and 1 µg wtSPINK6 plasmid or vector in BHK21 cells, culture media were replaced with DMEM medium supplemented with 5 µg/ml TPCK trypsin and incubated at 37°C for 30 min. We then lysed the cells in RIPA lysis buffer for Western blot analysis to examine HA cleavage pattern. Alternatively, at 48 h after transfection of 1 µg H7 plasmid, culture media were replaced with DMEM supplemented with 5 µg/ml TPCK trypsin, and 4 µg/ml recombinant wtSPINK6 or PBS or 1 µg/ml AEBSF (Merck Millipore). After incubation at 37°C for 30 min, the cells were then harvested for Western blot analysis using an α‐H_7_ antibody. Intensities of HA_2_ and HA_0_ bands were quantified with ImageJ, and the relative band intensity of HA_2_ versus that of HA_0_ was calculated.

We detected the proteolytic cleavage of H1 and H7 by various serine proteases. BHK21 cells seeded in a 24‐well plate were co‐transfected with either 1 µg H7 or H1 plasmid, and 500 ng plasmid encoding TMPRSS2 or HAT or Matriptase, or 1 µg KLK5 plasmid or Furin plasmid. At 48 h post‐transfection, we harvested the cells in RIPA lysis buffer to examine HA cleavage by Western blot analysis. The cells transfected with H7 or H1 plasmid only were incubated for 30 min in the presence or absence of 5 µg/ml TPCK trypsin, and applied to Western blot as a positive and negative control for HA cleavage, respectively. We evaluated SPINK6 inhibition of H7 and H1 cleavage mediated by various proteases. A triple transfection was performed, including 1 µg wtSPINK6 plasmid or mutSPINK6 or blank vector together with the above HA and protease plasmids. At 48 h post‐transfection, the transfected cells were harvested for Western blot analysis to detect HA cleavage using antibodies specific for the respective HA.

### Viral replication experiments in cell lines and human organoids

At 24 h post‐transfection of 1 µg wtSPINK6 vector or blank vector, A549 cells in a 24‐well plate were inoculated with H7N9/ah or H1N1/pdm at the indicated multiplicity of infection (MOI) and incubated in DMEM medium in the presence of 0.5 µg/ml TPCK trypsin. At the indicated hours post‐infection, infected cells and cell‐free culture medium (supernatant) were harvested for detection of viral load using RT‐qPCR assay as we described previously (Zhou *et al*, 2019). Alternatively, at 24 h post‐transfection of 2.5 µg HAT or KLK5 plasmid, A549 cells seeded in a 12‐well plate were inoculated with H1N1/pdm virus at a MOI of 0.25, and then incubated in DMEM medium in the presence of 20 µg/ml wtSPINK6 protein or mutSPINK6 protein. Cell‐free culture media were harvested at 24 hpi and stored in two aliquots; one aliquot was directly applied to the conventional plaque assay; and the other was treated with 2 µg/ml TPCK‐trypsin at 37°C for 1 h prior to the plaque assay.

BHK21 cells in a six‐well plate were co‐transfected in triplicate with 1.5 µg wtSPINK6 or blank vector with various protease plasmids including 1 µg TMPRSS2 plasmid or 1 µg HAT plasmid or 500 ng Matriptase plasmid. Cell‐free media were collected at 36 h post‐transfection from each well. The transfected cells were inoculated with H7N9/ah virus with a MOI of 0.5, and then incubated with the medium collected from the same well before inoculation. At 24 h post‐infection, cell‐free media were harvested and applied to plaque assay.

After ethical approval by the Institutional Review Board of the University of Hong Kong/Hospital Authority Hong Kong West Cluster (UW13‐364), human lung organoids were previously established using lung tissues adjacent to the diseased tissues from patients who underwent surgical resection as described previously (Zhou *et al*, 2018). The informed consent was obtained from all patients and that the experiments conformed to the principles set out in the WMA Declaration of Helsinki and the Department of Health and Human Services Belmont Report. At 24 h after inoculation of H1N1/pdm or mock inoculation in triplicate with a MOI of 0.1, 2D human airway organoids were harvested for detecting mRNA expression levels of the indicated serine proteases. Alternatively, 2D human airway organoids were inoculated with H1N1/pdm from the apical side with a MOI of 0.1, and then incubated in proximal differentiation medium for 24 h in the presence of 60 µg/ml wtSPINK6 protein or PBS in triplicate, or 20 µg/ml α‐SPINK6 or isotype rabbit IgG in triplicate. Cell‐free media were then collected for viral titration by plaque assay and detection of viral load by RT‐qPCR, and Western blot analysis to examine HA cleavage with a mouse antiserum collected from mice that survived a sublethal H1N1 virus infection after the media were concentrated by 10‐fold.

### Immunofluorescence staining and flow cytometry analysis

Immunofluorescence staining was performed to detect endogenous SPINK6 in human lung tissues and airway organoids. Briefly, after fixation with 4% PFA, permeabilization, and blocking, lung tissues and 2D human airway organoids were incubated with α‐SPINK6 (Abcam, ab110830) in Antibody Diluent buffer (Dako) overnight at 4°C, then followed with secondary antibody staining for 1 h at room temperature. Nuclei and actin filaments were counterstained with 4’,6‐diamino‐2‐phenylindole (DAPI) (Thermo Fisher Scientific) and Phalloidin‐647 (Sigma Aldrich), respectively. Mouse lung tissue slides were stained with antinucleoprotein of IAV (NP, Novus NBP2‐16965) to label virus‐infected cells. Confocal imaging was performed using a Carl Zeiss LSM 800 confocal microscope.

Flow cytometry analysis was performed to inspect 2D human airway organoids. In brief, the airway organoids were dissociated into single‐cell suspension after incubation in 10 mM EDTA (Invitrogen) for 60 min at 37°C. After PFA fixation, and permeabilization, the cells were incubated with primary antibodies α‐SPINK6 (Abnova, H00404203‐M04) and α‐HAT (Thermo Fisher Scientific, PA5‐42876) for 1 h at 4°C followed by staining with secondary antibodies. A BD FACSCanto II system was applied to examine the labeled cells. Data were analyzed using FlowJo v10 (Tree Star, USA)

### Plaque assay

Plaque assay was performed to determine titers of the virus stocks and supernatant samples as described elsewhere with minor modification (Zhou *et al*, 2017, 2018). Briefly, MDCK cells were seeded in 12‐well plates. Confluent monolayers were inoculated with 400 µl of 10‐fold serial dilutions of samples and incubated for 1 h at 37°C. After removing the inoculum, the monolayers were overlaid with 1% LMP Agarose (Invitrogen) supplemented with MEM and 1 µg/µl TPCK‐treated trypsin and further incubated for 2–3 days. The monolayers were fixed with 4% PFA and stained with 1% crystal violet to visualize the plaque after removing the agarose plugs. Virus titer was calculated as plaque‐forming units (PFU) per milliliter.

### Mouse experiment

Female Balb/c mice of 6–8 weeks old were maintained in standard Biosafety level 2 animal laboratory and given access to standard pellet feed and water *ad libitum*. In all animal experiments, we followed the operating procedures approved by the Committee on the Use of Live Animals in Teaching and Research, the University of Hong Kong. The mice were randomly divided into two groups, and inoculated with 5 PFU of A/Hong Kong/415742Md/2009(H1N1)pdm09, a mouse‐adapted strain of pandemic H1N1 virus that caused a lethal infection due to robust viral propagation in the mouse lungs (Zhou *et al*, 2019). At 24 h before inoculation, and 8, 24, and 36 h post‐inoculation, two groups of mice were intranasally administered with 0.5 µg/µl wtSPINK6 protein or mutSPINK6 protein in 20 µl. The virus inoculation and the administration of protein solutions were performed after anesthesia by intraperitoneal injection of 70–100 mg/kg ketamine and 10–20 mg/kg xylazine. We performed a lower pfu inoculation (5 pfu per mouse) than the originally defined LD50 of 150 pfu (Zheng *et al*, 2010) since we simultaneously performed three times of intranasal administration of SPINK6 proteins. The multiple intranasal administration itself exacerbated the infection. Thus, much lower challenge doses should be given to the mice than those without such manipulation (Smee *et al*, 2012). A total of 10 mice treated with wtSPINK6 or mutSPINK6 were monitored daily for disease signs, body weight, and survival for 14 days. A body weight loss exceeding 25% was set as the humane endpoint. We prevent unnecessary suffering by euthanasia when an animal is in such a state that survival is not possible. Five mice treated with wtSPINK6 or mutSPINK6 were sacrificed at 3 and 4 days after the viral challenge. Half of the lung tissue was collected and homogenized for the quantification of viral growth, while the other half was fixed in 4% PFA for tissue processing and immunofluorescence staining.

### Statistics analysis

Student’s *t*‐test analysis was used for data analysis, unless stated otherwise. In the mouse experiment, 10 mice in each group for monitoring survival and 5 biological replicates at each time point for assessing infection are previously determined as sufficient for a correct statistical assessment. The mice were randomly grouped to minimize the subjective bias. The immunofluorescence staining slides of mouse tissues were masked before an experienced pathologist reviewed the slides. Mouse survival data were analyzed with Mantel–Cox test. The statistical analysis was conducted with GraphPad Prism (version 8.0.1). A *P* < 0.05 is regarded as statistically significant.

## Author contributions

JZ designed the study. DW, CL, MCC, XL, XZ, YY, JH, SY, VP, and J‐PC performed the experiments. DW, JZ, ZC, HC, JF‐WC, and KKWT analyzed the data. JZ and KYY wrote the manuscript and provided grant support.

## Conflict of interest

J.Z., K.Y.Y., M.C.C., and C.L. are listed as inventors on the patent of airway organoids (publication No: US‐2021‐0207081‐A1). All other authors declare no competing interests.

## Supporting information



Expanded View Figures PDF

Source Data for Expanded View

Source Data for Figure 1

Source Data for Figure 2

Source Data for Figure 4

## Data Availability

This study includes no data deposited in external repositories.
