# Supplementary material for: SPINK6 inhibits human airway serine proteases and restricts influenza virus activation
Source: EMBO Mol Med. 2021 Nov 26;14(1):e14485. doi: 10.15252/emmm.202114485 (PMC9976594; doi:10.15252/emmm.202114485)
Supplement: Supplementary file 1 — Expanded View Figures PDF [file EMMM-14-e14485-s003.pdf]

Expanded View Figures

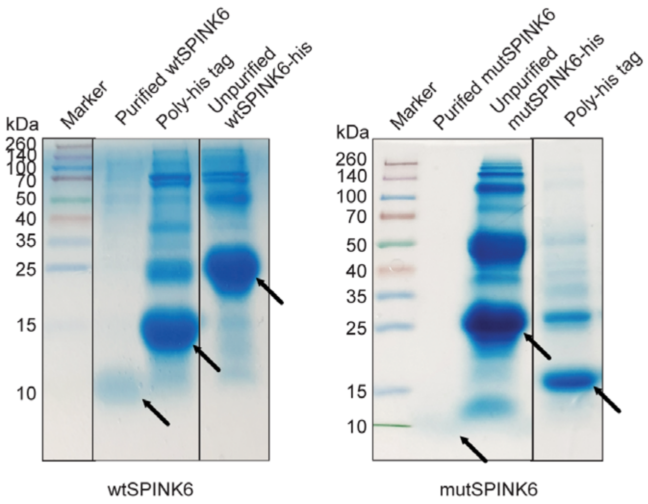

Figure EV1. Coomassie blue staining shows the raw and purified recombinant proteins..

Raw recombinant proteins of wtSPINK6 and mutSPINK6, purified wtSPINK6 and mutSPINK6 proteins, and removed poly-His tag are indicated with arrows after SDS-PAGE and Coomassie blue staining.

Source data are available online for this figure.

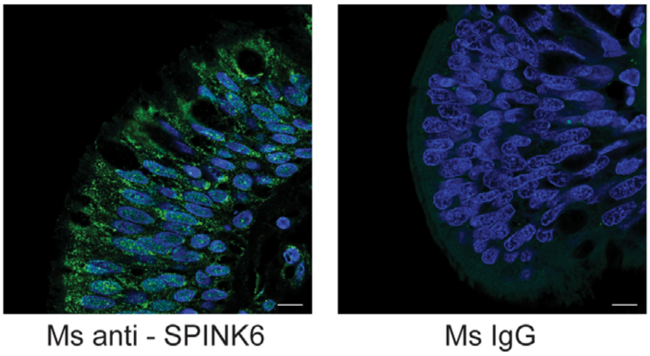

Figure EV2. SPINK6 is expressed in the airway epithelial cells in human bronchial tissue..

Paraffin slides of human lung tissues are stained with an α-SPINK6 (green, left) or an isotopic IgG (right) and applied to confocal imaging. Nuclei are counterstained with DAPI (blue). Scale bar, 10 μm.

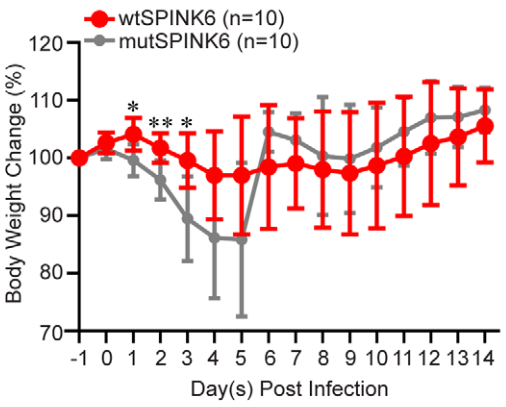

Figure EV3. SPINK6 treatment alleviated body weight loss in IAV-infected mice..

After IAV inoculation, body weights of 10 mice treated with wtSPINK6 or mutSPINK6 were monitored. Daily body weigh changes in survived mice are presented. Data represent mean ± SD. *$P < 0.05$; **$P < 0.01$. Student's *t*-test is used for data analysis.

WILEY

© 2021 The Authors

© 2021 The Authors
