# Supplementary figures and images for: SPINK6 inhibits human airway serine proteases and restricts influenza virus activation
Source: EMBO Mol Med. 2021 Nov 26;14(1):e14485. doi: 10.15252/emmm.202114485 (PMC9976594; doi:10.15252/emmm.202114485)

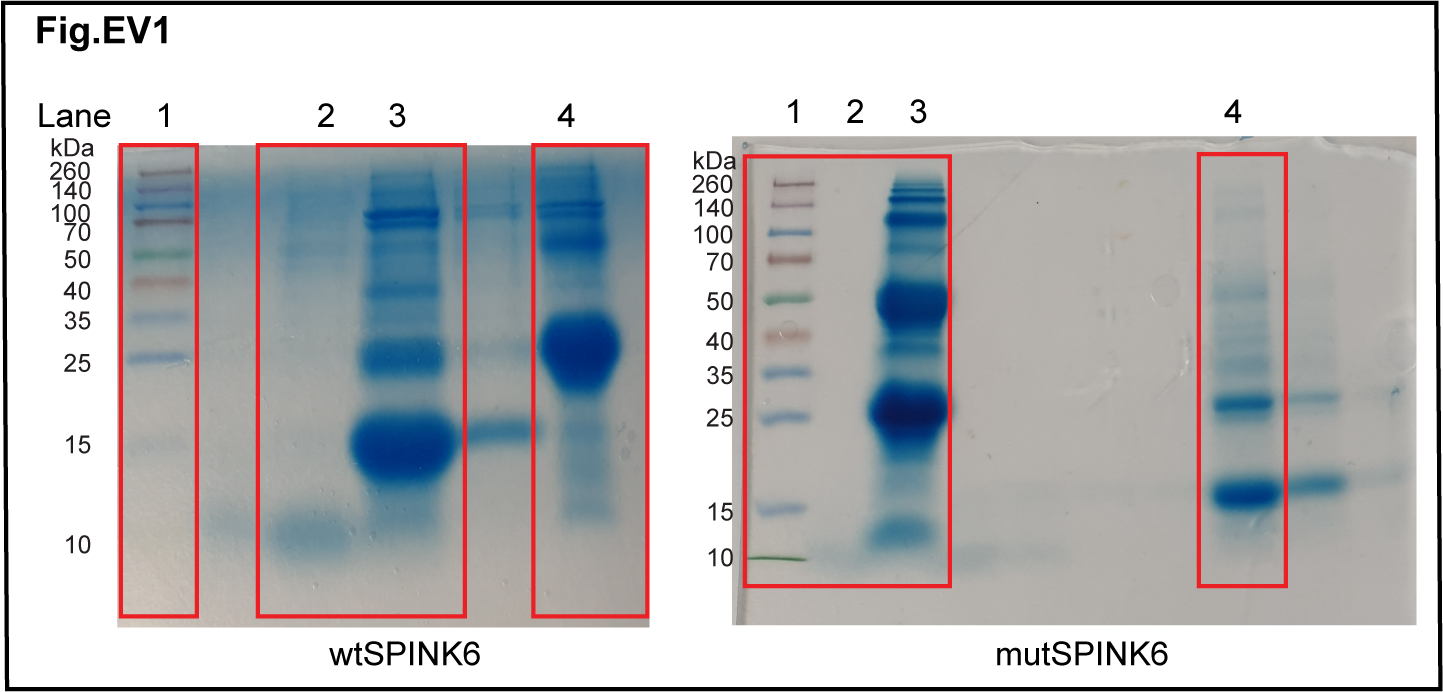

Supplement: Supplementary file 2 — Source Data for Expanded View [file EMMM-14-e14485-s006.zip › emmm202114485-sup-0005-SDataFigEV/emmm202114485-sup-0005-SDataFigEV.tif]

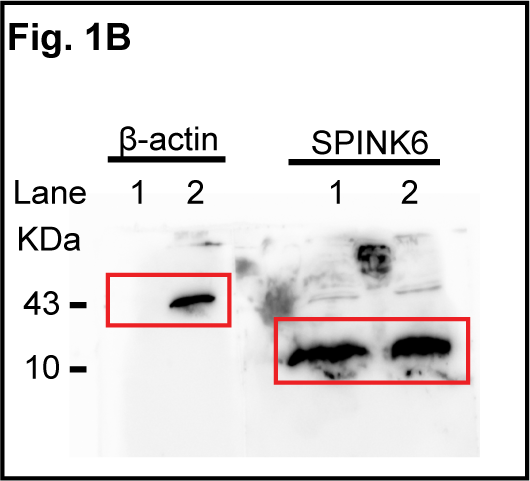

Supplement: Supplementary file 3 — Source Data for Figure 1 [file EMMM-14-e14485-s001.zip › Figure_1B.tif]

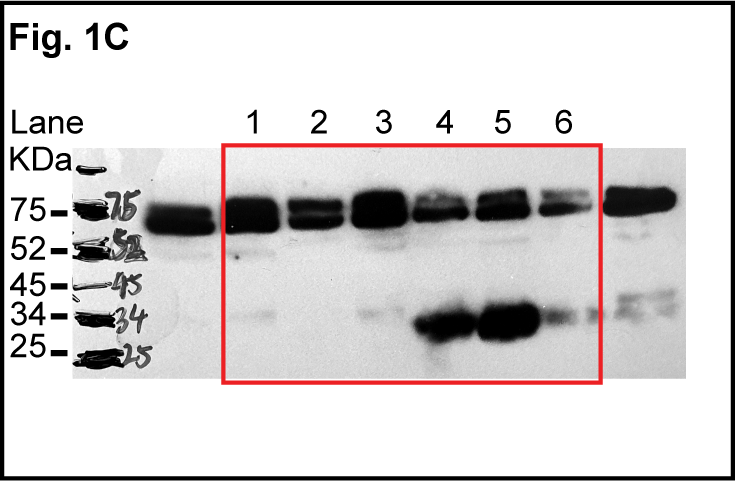

Supplement: Supplementary file 3 — Source Data for Figure 1 [file EMMM-14-e14485-s001.zip › Figure_1C.tif]

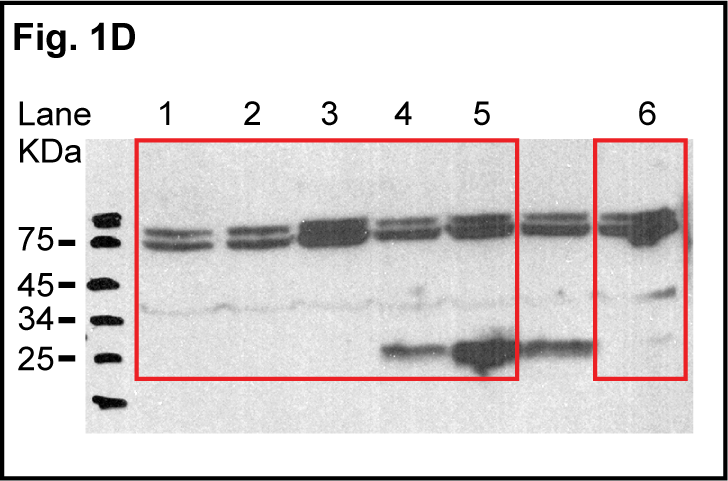

Supplement: Supplementary file 3 — Source Data for Figure 1 [file EMMM-14-e14485-s001.zip › Figure_1D.tif]

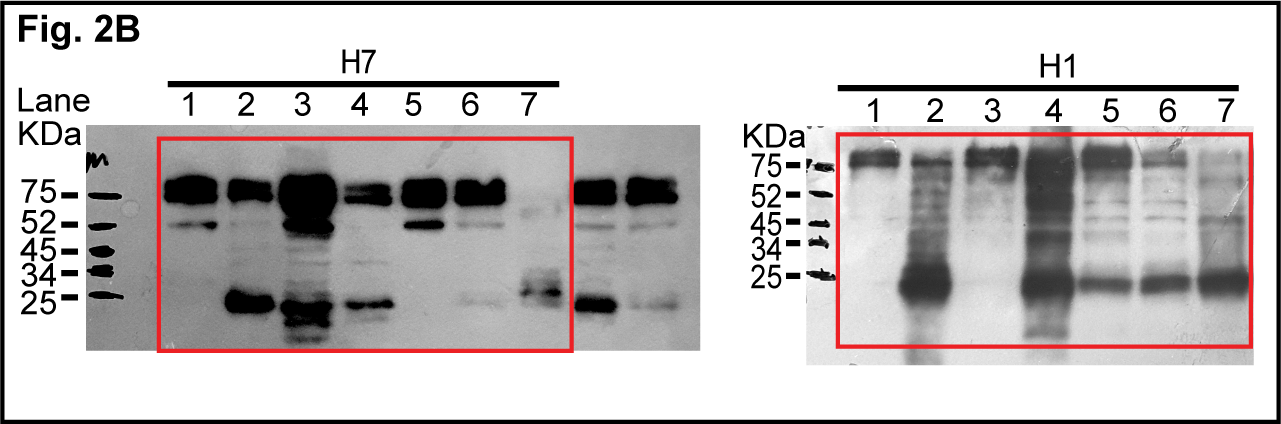

Supplement: Supplementary file 4 — Source Data for Figure 2 [file EMMM-14-e14485-s005.zip › Figure_2B.tif]

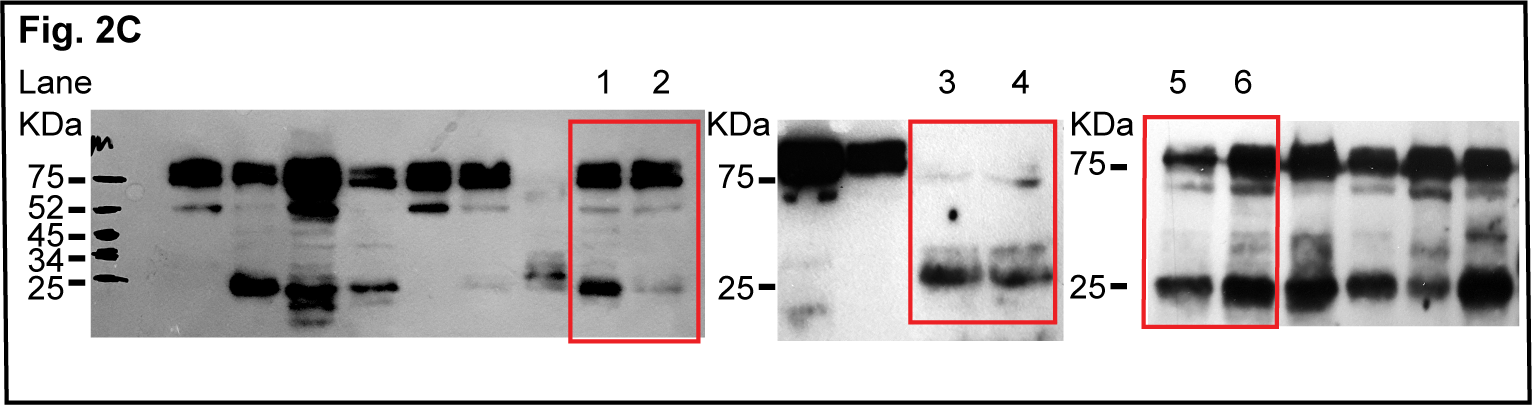

Supplement: Supplementary file 4 — Source Data for Figure 2 [file EMMM-14-e14485-s005.zip › Figure_2C.tif]

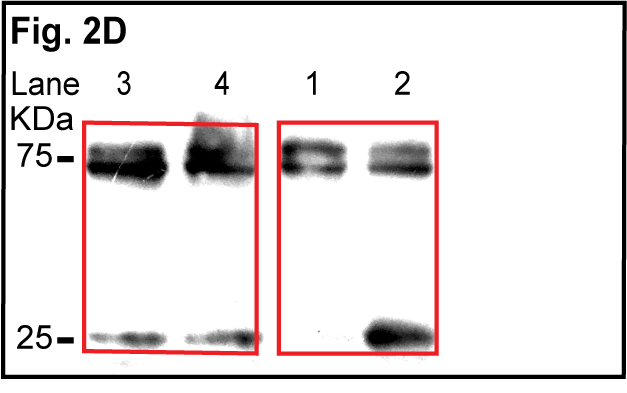

Supplement: Supplementary file 4 — Source Data for Figure 2 [file EMMM-14-e14485-s005.zip › Figure_2D.tif]

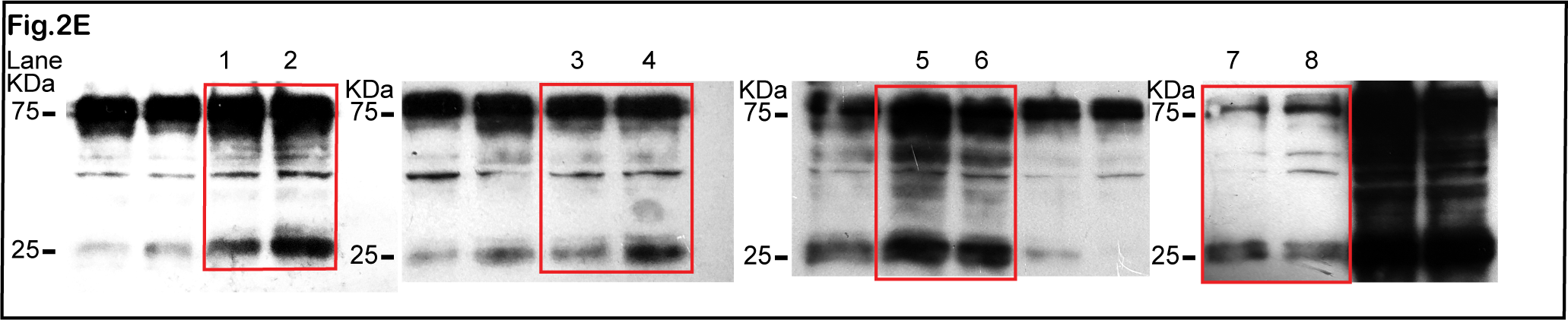

Supplement: Supplementary file 4 — Source Data for Figure 2 [file EMMM-14-e14485-s005.zip › Figure_2E.tif]

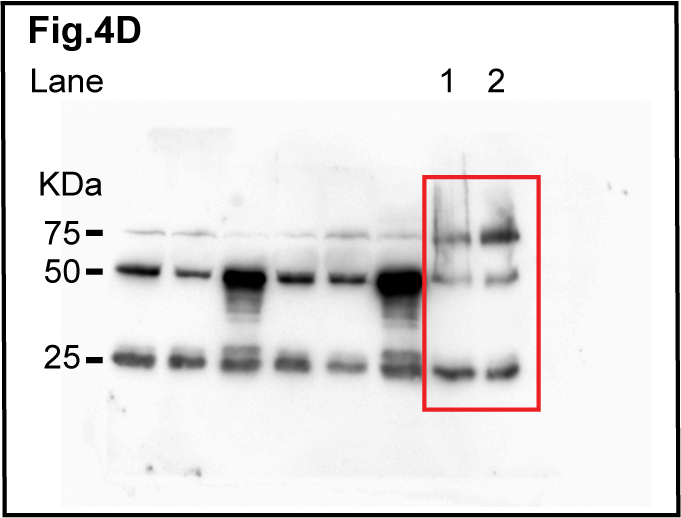

Supplement: Supplementary file 5 — Source Data for Figure 4 [file EMMM-14-e14485-s004.zip › Figure_4D.tif]

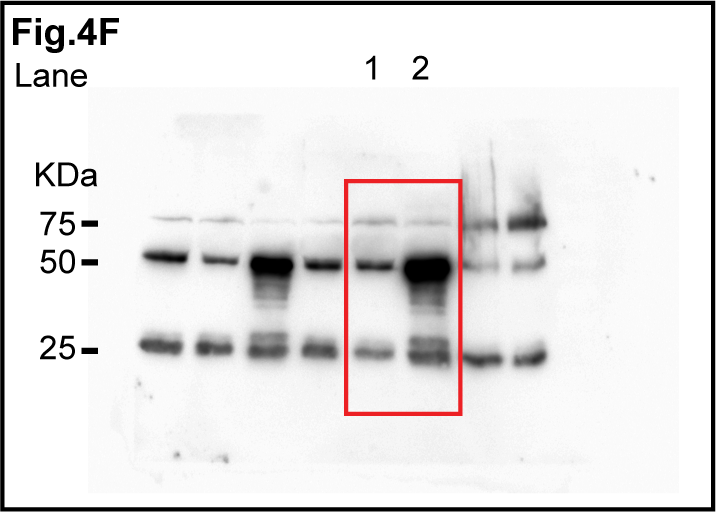

Supplement: Supplementary file 5 — Source Data for Figure 4 [file EMMM-14-e14485-s004.zip › Figure_4F.tif]
