# Supplementary material for: SPINK6 inhibits human airway serine proteases and restricts influenza virus activation
Source: EMBO Mol Med. 2021 Nov 26;14(1):e14485. doi: 10.15252/emmm.202114485 (PMC9976594; doi:10.15252/emmm.202114485)
Supplement: Supplementary file 6 [file EMMM-14-e14485-s002.pdf]

EMBO Molecular Medicine

SPINK6 inhibits human airway serine proteases and restricts influenza virus activation

Dong Wang, Cun Li, Man Chiu, Yifei YU, Xiaojuan Liu, Xiaoyu Zhao, Jingjing Huang, Zhongshan Cheng, Shuofeng Yuan, Vincent Poon, Jian-Piao Cai, Hin Chu, Jasper Fuk-Woo Chan, Kelvin To, Kwok-Yung Yuen, and Jie Zhou
DOI: 10.15252/emmm.202114485

Corresponding author: Jie Zhou (jiezhou@hku.hk)

Review Timeline:

Submission Date:	14th Jan 21
Editorial Decision:	3rd Feb 21
Revision Received:	28th Apr 21
Editorial Decision:	18th May 21
Revision Received:	11th Oct 21
Editorial Decision:	26th Oct 21
Revision Received:	6th Nov 21
Accepted:	10th Nov 21

Editor: Zeljko Durdevic

Transaction Report:

(Note: With the exception of the correction of typographical or spelling errors that could be a source of ambiguity, letters and reports are not edited. Depending on transfer agreements, referee reports obtained elsewhere may or may not be included in this compilation. Referee reports are anonymous unless the Referee chooses to sign their reports.)

Dear Dr. Zhou,

Thank you for the submission of your manuscript to EMBO Molecular Medicine. We have now received feedback from the three reviewers who agreed to evaluate your manuscript.

As you will see from their reports pasted bellow, while the referee #2 is overall supportive of the study, the referees #1 and #3 acknowledge the interest of the study, however they also have serious and partially overlapping concerns that preclude further consideration of the article at this time. Given the nature of these criticisms, addressing all the referees' comments would require a lot of additional work, time and effort. As clear and conclusive insight into a novel clinically relevant observation is key for publication in EMBO Molecular Medicine, I am afraid that we do not feel it would be productive to call for a revised version of your manuscript at this stage and therefore we cannot offer to publish it.

Given the potential interest and novelty of the findings, we would, however, be willing to consider a new manuscript on the same topic if at some time in the near future you obtained data that would considerably strengthen the message of the study and address the referees concerns in full. To be completely clear, however, I would like to stress that if you were to send a new manuscript this would be treated as a new submission rather than a revision and would be reviewed afresh, in particular with respect to the literature and the novelty of your findings at the time of resubmission. If you decide to follow this route, please make sure you nevertheless upload a letter of response to the referees' comments.

I am sorry that I could not bring better news this time and hope that the referee comments are helpful in your continued work in this area.

Yours sincerely,

Zeljko Durdevic

Zeljko Durdevic
Editor
EMBO Molecular Medicine

***** Reviewer's comments *****

Referee #1 (Comments on Novelty/Model System for Author):

1. The initial findings were based on human polymorphisms in the SPINK6 gene. While these were interesting data to present, experiments performed subsequently did not consider the impact of these polymorphisms on SPINK6 function. Rather, the mutated SPINK6 used in this study contained a loss-of-function R19A mutation. If this change correlates with the rs1432689 SNP, the authors should indicate this more clearly. I do not have extensive experience in human genetics, so please let me know if there is something that I am missing here. I basically want to determine if the human connection between the GWAS study and the experiments that were subsequently performed are related.

2. In the flow cytometry data presented in Figure 5, would it be possible for the authors to provide the individual histograms for the fluorescent channels as well as the plots showing the co-staining in 4 quadrants. It was difficult to see where the populations were in the plots presented, making it unclear how it was determined that 30% of the cells in the organoids expressed SPINK6.
3. The cell culture studies presented variable MOIs for inoculation, making it difficult to compare some of the infections across the full manuscript.
4. In the murine study, would the authors be willing to describe how they decided the dose and delivery for SPINK6. While the survival data were interesting, and supported the hypothesis, the weight loss and lung titer data did not match well with survival results. Specifically, with the large error bars in the weight loss data, I wonder if there were two different responses within the challenge groups with some mice losing more weight than others. If this is the case, the authors should clearly present these differences in response to the virus infection.
5. Further, the virus titers were quite high in both groups, indicating that SPINK6 does not effectively eliminate the virus infection. Would a different dose of SPINK6 have a greater effect on virus infectivity?
6. Overall, as presented, it is not clear that the conclusions from the results presented fully support the title which implies that SPINK6 has a much greater effect on virus propagation in living systems, especially in humans.

Referee #1 (Remarks for Author):

Comments for the authors of the EMBO Molecular Medicine manuscript number EMM-2021-13691:
The authors of the EMBO Molecular Medicine manuscript "SPINK6 inhibits human airway serine proteases and restricts influenza virus activation", present their findings related to the impact of proteases and anti-proteases on influenza hemagglutinin cleavage and infection. Specifically, they focus on the serine protease inhibitor Kazal-type 6 (SPINK6), human data that correlates genetic variation in SPINK 6 with susceptibility to H7N9 infection. The authors then proceed to evaluate HA cleavage from HA0 into HA2 in the presence of known proteases, using SPINK6 as an anti-protease. They draw the conclusion that SPINK6 inhibits HAT and KLK5 protease activity, which restricts HA cleavage and is associated with reduced infectivity in human organoid and murine infection models. Their overall conclusion is that SPINK6 is an inhibitor of serine proteases in a manner that restricts influenza A virus infection in the human respiratory tract. While these findings interesting, and the experimental progression both logical and well-designed, I have identified come limitations to the study that will be presented below.
General Comments:

1. The initial findings were based on human polymorphisms in the SPINK6 gene. While these were interesting data to present, experiments performed subsequently did not consider the impact of these polymorphisms on SPINK6 function. Rather, the mutated SPINK6 used in this study contained a loss-of-function R19A mutation. If this change correlates with the rs1432689 SNP, the authors should indicate this more clearly. I do not have extensive experience in human genetics, so please let me know if there is something that I am missing here. I basically want to determine if the human connection between the GWAS study and the experiments that were subsequently performed are related.
2. In the flow cytometry data presented in Figure 5, would it be possible for the authors to provide the individual histograms for the fluorescent channels as well as the plots showing

the co-staining in 4 quadrants. It was difficult to see where the populations were in the plots presented, making it unclear how it was determined that 30% of the cells in the organoids expressed SPINK6.

3. The cell culture studies presented variable MOIs for inoculation, making it difficult to compare some of the infections across the full manuscript.
4. In the murine study, would the authors be willing to describe how they decided the dose and delivery for SPINK6. While the survival data were interesting, and supported the hypothesis, the weight loss and lung titer data did not match well with survival results. Specifically, with the large error bars in the weight loss data, I wonder if there were two different responses within the challenge groups with some mice losing more weight than others. If this is the case, the authors should clearly present these differences in response to the virus infection.
5. Further, the virus titers were quite high in both groups, indicating that SPINK6 does not effectively eliminate the virus infection. Would a different dose of SPINK6 have a greater effect on virus infectivity?
6. Overall, as presented, it is not clear that the conclusions from the results presented fully support the title which implies that SPINK6 has a much greater effect on virus propagation in living systems, especially in humans.

Specific comments

1. There were numerous times in the reading of the manuscript where the sentence structure and grammar could be improved.
2. In Figure 2D, it was not entirely clear that the wtSPINK6 was provided as a recombinant protein. Please make this clear within the figure.
3. Large portions of the Discussion section simply repeated the results rather than presenting them in the context of the field.

Referee #2 (Remarks for Author):

The authors have identified in this study a serine protease inhibitor, SPNK6, that interferes with proteolytic activation of influenza A viruses of subtypes H7 and H1. They show that SPNIK6 inhibits trypsin-mediated cleavage of virus replication in vivo and blocks activation by the airway-specific proteases HAT and KLK5. Interestingly, it does not inhibit TMPRSS2 and matriptase, other serine proteases also known to activate H7 and H1 influenza viruses. Furthermore, it is shown that SPINK6 is expressed intrinsically in human airway organoids where it also interferes with virus replication. Similar observations have been made in a mouse model. These are very interesting observations. It has long been known that ptoteilytic activation which is an important determinant of tissue tropism, host range and pathogenicity of influenza and many other viruses depends on the structure of the viral substrate, in this case the hemagglutinin, and the substrate specificity of the host protease. From the present study it is now clear that the specificity of proteolytic activation depends also on a third group of factors, protease inhibitors provided by the host.

Points of objection.

Lines 109-111: The authors refer here to a study by Chen et al., 2015, which shows that galectin regulates susceptibility to H7N9 infection. According to the authors, this observation suggests an involvement of SPINK6 in protection from virus infection. However the link between galectin and SPINK6 remains unclear. This link has to be explained..

Line 161: Fig.2B should be Fig.2C.

Referee #3 (Comments on Novelty/Model System for Author):

No significant conclusions can be drawn from the animal experiments for several reasons:

1. Very low animal numbers are used not allowing any significant conclusion.
2. Virus infectious dose of 5 pfu is far too low for reproducible infection. The results can be simply due to the variations in viral inoculation efficiency.
3. The animal protocol is not well described (e.g. humane endpoints are not defined).

Referee #3 (Remarks for Author):

The study by Wang et al. describes a previously reported inhibitor of serine protease families, namely SPINK6. The authors show that SPINK6 inhibits proteolytic cleavage of influenza HA and inhibits viral gene expression. The authors further explored the therapeutic potential of SPINK6 in mice. While some observations are interesting, the study is at a very premature stage. Following major concerns dampen my enthusiasm:

1. Database analysis reveal according to the authors a potential role for SPINK6 SNPs in human H7N9 infection by increased transcription. This is a potential important findings that needs to be experimentally validated. Data shown in Figure 1 are in silico data. The authors should use human biopsy material and assess SPINK6 expression in relation to key proteases, such as HAT or TMPRSS2 in the upper and lower respiratory tract. Also, they should study whether viral infection affects SPINK6 mRNA expression that could have major implications on the infection course.

2. There are no data showing that SPINK6 actually inhibits H7N9 replication. In Figure 2E only vRNA amounts are shown. The authors should show infectious virus titres as p.f.u. over several time points post infection.

3. Animal data shown in Figure 6 are not allowing any conclusions. The authors have used a virus infectious dose of 5 p.f.u., which is too low to allow reproducible animal infection yet any meaningful conclusions. It is not clear why the authors used H1N1 influenza instead of H7N9 that is the primary focus of the study. It was repeatedly shown that H7N9 is able to infect mice and cause weight loss. The authors have used only 4 mice per time point assessed in Figure 6c. This is statistically not sufficient to allow significant conclusions. Further, the animal protocol is poorly described in the M&M section. There is no description of the narcosis used for infection and treatment. Humane endpoints are not defined.

Referee #1 (Comments on Novelty/Model System for Author):

1. The initial findings were based on human polymorphisms in the SPINK6 gene. While these were interesting data to present, experiments performed subsequently did not consider the impact of these polymorphisms on SPINK6 function. Rather, the mutated SPINK6 used in this study contained a loss-of-function R19A mutation. If this change correlates with the rs1432689 SNP, the authors should indicate this more clearly. I do not have extensive experience in human genetics, so please let me know if there is something that I am missing here. I basically want to determine if the human connection between the GWAS study and the experiments that were subsequently performed are related.

A: We appreciate the reviewer's comment. The integrative analysis of genetic association and eQTL suggested that higher SPINK6 expression may confer protection from human H7N9 infection. In combination with other biological evidence described in the introduction section, we formulated the hypothesis that SPINK6 may inhibit common HA cleavage serine proteases, besides the previously characterized targets KLK5 and KLK12. In the following experiments, we've used the conventional gain-of-function and/or loss-of-function experiments to demonstrate the role of SPINK6 for HA cleavage and IAV replication mediated by serine proteases. The mutant SPINK6 with a defective protease inhibition domain has nothing to with SNP rs1432689. The association SNP rs1432689 is not involved in protein encoding, instead it is correlated to the differential mRNA expression levels of SPINK6 (Figure 6). The possible genetic basis for the correlation has been described in the text (page 10 line 280 in the current manuscript).

Thanks to the reviewer's comment, we realize that data presentation in the original manuscript was inappropriate so that the reviewer may be confused. In fact, the other biological evidence we have gleaned from previous studies (mentioned in the introduction section) is sufficient to formulate the hypothesis, without the results of the human genetic association studies. Therefore, we re-organized the data presentation. In the revised manuscript, the genetic association and eQTL are presented at the end of the results as a supportive evidence in humans. This is actually the most common way of data presentation in many similar studies.

2. In the flow cytometry data presented in Figure 5, would it be possible for the authors to provide the individual histograms for the fluorescent channels as well as the plots showing the co-staining in 4 quadrants. It was difficult to see where the populations were in the plots presented, making it unclear how it was determined that 30% of the cells in the organoids expressed SPINK6.

A: We amended the figure of flow cytometry results (Figure 4A) as suggested by the reviewer. The y axis and x axis represent the expression of HAT and SPINK6 respectively. The quadrants are set to delineate HAT+, SPINK6+ and the negative populations, the number in each quadrant represents the percentage of cells within the quadrant. Hopefully, we have addressed the reviewer's inquiry.

3. The cell culture studies presented variable MOIs for inoculation, making it difficult to compare some of the infections across the full manuscript.

A: We have designed various experiments to demonstrate the role of SPINK6 for virus replication driven by exogenous proteases (overexpression of selected proteases or the addition of trypsin in culture media) or endogenous proteases (in airway organoids). In these experiments, we have to optimize various elements, which are very distinct in different experimental settings. Overall, we aimed to achieve an active viral growth under various settings, then examined the effect of SPINK6 on viral growth. For example, in infection experiments in cell lines, the addition of TPCK-trypsin (Figure 1E) enabled HA activation with a higher potency/efficiency than overexpression of selected proteases (Figure 3A), which is well expected. Thus, a lower MOI inoculation was done in the former than the latter. Despite distinct MOIs in different experimental settings, we believe the same conclusion has been reached, i.e., SPINK6 inhibits HAT- and KLK5-mediated HA cleavage and viral growth.

4. In the murine study, would the authors be willing to describe how they decided the dose and delivery for SPINK6. While the survival data were interesting, and supported the hypothesis, the weight loss and lung titer data did not match well with survival results. Specifically, with the large error bars in the weight loss data, I wonder if there were two different responses within the challenge groups with some mice losing more weight than others. If this is the case, the authors should clearly present these differences in response to the virus infection.

A: We appreciate the reviewer's comments. Firstly, we chose to deliver SPINK6 protein by intranasal administration since it is a more effective delivery route to reach the infection site (mouse lung) than other routes such as intra-peritoneal injection or intra-venous injection. In addition, due to the challenge of respiratory delivery, we performed pilot experiments with various doses of protein solutions. Based on the results, we elected an experimental scheme of multiple dosing (3 times) with 10ug protein in a volume of 20ul. Under this setting, two groups of mice appeared tolerated to the administration and displayed indistinguishable response right after the manipulation. Moreover, we have demonstrated in the *in vitro* experiments that the addition of SPINK6 protein onto 2D airway organoids can inhibit protease activity, HA cleavage and viral growth (Figure 4). Hence, the intranasal administration of SPINK6 was performed as an equivalent manipulation in mice. We have revised the manuscript accordingly on page 9.

We agree with the reviewer on the big error bar in body weight data. We believe it is related to the experimental design. Apart from the virus inoculation, we performed intranasal delivery of wildtype and mutant SPINK6 proteins for 3 times. The multiple dosing exerted additional stress on these virus-infected mice and exacerbated the infection in both groups, some of which may lose more body weight than the others, leading to a big variation in the body weight. In the mutant SPINK6 treatment group, the mice developed more severe disease and lost more body weight with 7 deaths on day 4 ~ day 6, only 3 mice survived the infection. Accordingly, the variation of body weight was even bigger on day 3 ~ day 5 and it became smaller from day 6 when the heavily-sick mice had died. We have described different manifestations of two groups of mice on page 9 in the revised manuscript, as suggested by the reviewer.

5. Further, the virus titers were quite high in both groups, indicating that SPINK6 does not

effectively eliminate the virus infection. Would a different dose of SPINK6 have a greater effect on virus infectivity?

A: We agree with the reviewer that SPINK6 did not eliminate the infection. we'd like to emphasize that, in this experiment, 3-time intranasal administration of protein solutions itself was quite harsh, and aggravated the viral infection. For mice of 6~8 week-old, we have used the maximal dose and volume tolerable to the mice based on our pilot experiments. The aim of the mouse experiment is to verify the effect of SPINK6 demonstrated in vitro. Despite the failure to eliminate virus infection by SPINK6 treatment, we may have adequately fulfilled the aim. A more effective and less invasive approach of delivery is definitely required for developing SPINK6 as an effective therapeutics against influenza.

6. Overall, as presented, it is not clear that the conclusions from the results presented fully support the title which implies that SPINK6 has a much greater effect on virus propagation in living systems, especially in humans.

A: We respectfully disagree with the comment. Probably our suboptimal data presentation in the previous manuscript was unable convince the reviewer. We hope the reviewer would appreciate the revised manuscript.

Referee #2 (Remarks for Author):

The authors have identified in this study a serine protease inhibitor, SPNK6, that interferes with proteolytic activation of influenza A viruses of subtypes H7 and H1. They show that SPNIK6 inhibits trypsin-mediated cleavage of virus replication in vivo and blocks activation by the airway-specific proteases HAT and KLK5. Interestingly, it does not inhibit TMPRSS2 and matriptase, other serine proteases also known to activate H7 and H1 influenza viruses. Furthermore, it is shown that SPINK6 is expressed intrinsically in human airway organoids where it also interferes with virus replication. Similar observations have been made in a mouse model. These are very interesting observations. It has long been known that ptoteilytic activation which is an important determinant of tissue tropism, host range and pathogenicity of influenza and many other viruses depends on the structure of the viral substrate, in this case the hemagglutinin, and the substrate specificity of the host protease. From the present study it is now clear that the specificity of proteolytic activation depends also on a third group of factors, protease inhibitors provided by the host.

A: We appreciate the reviewer's insightful and encouraging comments.

Points of objection.

Lines 109-111: The authors refer here to a study by Chen et al., 2015, which shows that galectin regulates susceptibility to H7N9 infection. According to the authors, this observation suggests an involvement of SPINK6 in protection from virus infection. However the link between galectin and SPINK6 remains unclear. This link has to be explained.

A: We cited the paper (Chen, Zhou et al. 2015), in which the original GWAS was described and the susceptibility gene galectin was characterized. Based on further data mining in the original GWAS and integrative analysis with eQTL datasets, we formulated the hypothesis of

SPINK6 and conducted this study. Hence, galectin is nothing to do with SPINK6. However, after considering the comments of the other reviewers, we found this part of data is actually dispensable for formulating the hypothesis, instead it seems to cause a biased interpretation. We decide to change the data presentation and present them at the end of results.

Line 161: Fig.2B should be Fig.2C.

A: We have corrected the mistake.

Referee #3 (Remarks for Author):

The study by Wang et al. describes a previously reported inhibitor of serine protease families, namely SPINK6. The authors show that SPINK6 inhibits proteolytic cleavage of influenza HA and inhibits viral gene expression. The authors further explored the therapeutic potential of SPINK6 in mice. While some observations are interesting, the study is at a very premature stage. Following major concerns dampen my enthusiasm:

1. Database analysis reveal according to the authors a potential role for SPINK6 SNPs in human H7N9 infection by increased transcription. This is a potential important findings that needs to be experimentally validated. Data shown in Figure 1 are in silico data. The authors should use human biopsy material and assess SPINK6 expression in relation to key proteases, such as HAT or TMPRSS2 in the upper and lower respiratory tract. Also, they should study whether viral infection affects SPINK6 mRNA expression that could have major implications on the infection course.

A: We appreciate the reviewer's constructive comments. In brief, we have conducted a genome-wide genetic association study in 2013. Through integrative analysis of the genetic association results and eQTL datasets, we found that the risk variants to H7N9 infection (association data) are correlated to the higher SPINK6 expression level in human lung tissues (eQTL data). In the previous manuscript, this part of data was presented as one of evidences to formulate the hypothesis. After considering the reviewer's comment, we realize the presentation of this part of the data is not satisfactory. Hence, we re-organized the data presentation. In the revised manuscript, the genetic association and eQTL are presented at the end of the results as a supportive evidence in humans. This is actually the most common way of data presentation in many similar studies. We hope the revised manuscript would present our findings more rationally and explicitly.

The experiments suggested by the reviewer are very important. However, human biopsy materials are not readily available for research purpose; this is the reason why many eQTL datasets are generated and given access to all researchers. Moreover, it is difficult to quantitatively assess SPINK6 expression in relation to key proteases, especially the dynamic interplay of SPINK6 and these proteases, in human tissues, since it is quite challenging to maintain the viability of human tissues during such an experiment. We are the team establishing the first airway organoid model (Zhou, Li et al. 2018). In these physiologically-active airway organoids, we demonstrate that influenza virus infection upregulates SPINK6; the addition of SPINK6 protein or antibody significantly modulates the activities of

endogenous proteases, HA cleavage and viral growth (the current Figure 4 or previous Figure 3). we hope the organoid data could adequately address the reviewer's inquiry.

2. There are no data showing that SPINK6 actually inhibits H7N9 replication. In Figure 2E only vRNA amounts are shown. The authors should show infectious virus titres as p.f.u. over several time points post infection.

A: We demonstrated SPINK6 suppression of trypsin-driven H7N9 replication by vRNA results (Figure 2E). Additional data with infectious virus titer was also presented. SPINK6 suppressed HAT-activated H7N9 replication by plaque assay in the previous figure 4A (current Figure 3A). We always use multiple assays to reach a conclusion, including this study.

3. Animal data shown in Figure 6 are not allowing any conclusions. The authors have used a virus infectious dose of 5 p.f.u., which is too low to allow reproducible animal infection yet any meaningful conclusions. It is not clear why the authors used H1N1 influenza instead of H7N9 that is the primary focus of the study. It was repeatedly shown that H7N9 is able to infect mice and cause weight loss. The authors have used only 4 mice per time point assessed in Figure 6c. This is statistically not sufficient to allow significant conclusions. Further, the animal protocol is poorly described in the M&M section. There is no description of the narcosis used for infection and treatment. Humane endpoints are not defined.

A: We appreciate the reviewer's comment, from which we recognized an insufficient description of mouse experiments in the previous manuscript. A more detailed description of the mouse experiments and justification of experimental design are provided in the revised manuscript on page 9 and page 17. In this study, we have used a mouse-adapted strain of H1N1pdm virus. We demonstrated previously that robust viral growth of the virus in lung tissues led to a fatal outcome in young female Balb/c mice (Zheng, Chan et al. 2010). Survival rate is normally the golden standard to demonstrate the effect of an intervention in similar mouse studies. To compare the survival rate, we have allotted 10 mice per group for wildtype and mutant SPINK6 treatment. The significantly higher survival rate in wildtype-SPINK6-treated mice than mutant-SPINK6-treated mice (80% versus 30%) lends strong support to our hypothesis that SPINK6 inhibits the activation and propagation of IAVs. For detection of viral load and viral titer in lung tissues, we had 4 mice in each group, which may not be a big sample size. Nonetheless, survival rate may provide a more comprehensive evaluation of mouse infection. Importantly, the survival rate data is very consistent to viral growth.

We agree with the reviewer that a virus inoculation of 5 pfu is quite low in most cases. However, we'd like to direct the reviewer's attention to the design of the mouse experiments. Apart from virus inoculation, we performed 3 times of intranasal administration of SPINK6 proteins. The multiple intranasal administration itself exacerbated the infection and promoted viral growth even after a low MOI inoculation. Nevertheless, we observed a significantly lower lung virus titer in mice treated with wildtype SPINK6 than those treated with the mutant protein.

In mouse experiments, we used a mouse-adapted strain of H1N1pdm virus (Zheng, Chan et al. 2010). First, H1N1 viruses can be handled in P2 animal lab, which is less demanding than handling H7N9 virus in P3 animal lab. Secondly, we aimed to verify the *in vitro* findings, i.e., SPINK6 inhibition of virus activation and growth driven by serine proteases. Two subtypes of virus used as the targets of serine proteases, H1N1 and H7N9, share similar proteases for HA cleavage. Both can be cleaved by HAT (Figure 2B), a major serine protease in the airway epithelium. As such, it doesn't matter which virus is used for the mouse experiment. We selected a mouse-adapted H1N1 strain, which is more readily handled in P2 lab. We'd like to emphasize that the primary focus of the study is SPINK6 inhibition of HA-activating proteases HAT and KLK5 rather than H7N9. These serine proteases can activate most influenza viruses including H1N1 and H7N9.

A: We thank the reviewer's comment for the description of mouse experiment, including euthanasia and the humane endpoint. We have amended the relevant part in materials and methods accordingly.

References

Chen, Y., J. Zhou, Z. Cheng, S. Yang, H. Chu, Y. Fan, C. Li, B. H. Wong, S. Zheng, Y. Zhu, F. Yu, Y. Wang, X. Liu, H. Gao, L. Yu, L. Tang, D. Cui, K. Hao, Y. Bosse, M. E. Obeidat, C. A. Brandsma, Y. Q. Song, K. K. To, P. C. Sham, K. Y. Yuen and L. Li (2015). "Functional variants regulating LGALS1 (Galectin 1) expression affect human susceptibility to influenza A(H7N9)." Sci Rep **5**: 8517.

Zheng, B., K. H. Chan, A. J. Zhang, J. Zhou, C. C. Chan, V. K. Poon, K. Zhang, V. H. Leung, D. Y. Jin, P. C. Woo, J. F. Chan, K. K. To, H. Chen and K. Y. Yuen (2010). "D225G mutation in hemagglutinin of pandemic influenza H1N1 (2009) virus enhances virulence in mice." Exp Biol Med (Maywood) **235**(8): 981-988.

Zhou, J., C. Li, N. Sachs, M. C. Chiu, B. H. Wong, H. Chu, V. K. Poon, D. Wang, X. Zhao, L. Wen, W. Song, S. Yuan, K. K. Wong, J. F. Chan, K. K. To, H. Chen, H. Clevers and K. Y. Yuen (2018). "Differentiated human airway organoids to assess infectivity of emerging influenza virus." Proc Natl Acad Sci U S A **115**(26): 6822-6827.

18th May 2021

Dear Dr. Zhou,

Thank you for the submission of your manuscript to EMBO Molecular Medicine. We have now heard back from the two referees who agreed to re-evaluate your manuscript. As you will see from the reports below, while the referee #1 is supporting publication of the study, referee #2 (previously #3) evaluated the revision as unsatisfactory particularly regarding your responses to the points #1 and #3. From the editorial side, we find you addressed the point #1 adequately, however, we agree with the referee #2 that the animal experiments are inconclusive.

Taking this in consideration it is clear that publication of the paper cannot be considered at this stage. I also note that addressing the reviewers concerns in full will be necessary for further considering the manuscript in our journal and this appears to require a lot of additional work and experimentation. I am unsure whether you will be able or willing to address those and return a revised manuscript within the six months deadline. On the other hand, given the potential interest of the findings, I would be willing to consider a revised manuscript with the understanding that the referee #2 (previously #3) concerns regarding animal experiments must be experimentally addressed and that acceptance of the manuscript would entail a second round of review.

Please note that EMBO Molecular Medicine encourages a single round of revision only and therefore, acceptance or rejection of the manuscript will depend on the completeness of your responses included in the next, final version of the manuscript. For this reason, and to save you from any frustrations in the end, I would strongly advise against returning an incomplete revision and would also understand your decision if you chose to rather seek rapid publication elsewhere at this stage.

I look forward to receiving your revised manuscript.

Should you find that the requested revisions are not feasible within the constraints outlined here and choose, therefore, to submit your paper elsewhere, we would welcome a message to this effect.

Yours sincerely,

Zeljko Durdevic

Zeljko Durdevic
Editor
EMBO Molecular Medicine

***** Reviewer's comments *****

Referee #1 (Remarks for Author):

Comments for the authors of the EMBO Molecular Medicine manuscript number EMM-2021-14485:
The authors of the EMBO Molecular Medicine manuscript "SPINK6 inhibits human airway serine proteases and restricts influenza virus activation", present their findings related to the impact of proteases and anti-proteases on influenza hemagglutinin cleavage and infection. They focus on the serine protease inhibitor Kazal-type 6 (SPINK6), and the authors evaluate HA cleavage from HA0 into HA2 in the presence of known proteases, using SPINK6 as an anti-protease. They draw the conclusion that SPINK6 inhibits HAT and KLK5 protease activity, which restricts HA cleavage and is associated with reduced infectivity in human organoid and murine infection models. They then present human data that correlates genetic variation in SPINK 6 with susceptibility to H7N9 infection. Their overall conclusion is that SPINK6 is an inhibitor of serine proteases in a manner that restricts influenza A virus infection in the human respiratory tract. These findings are interesting, and the data are presented in a manner that tells an interesting story that highlights the impact of their findings in the field.
General Comments:
1. This manuscript presents some interesting information that advances the field of viral pathogenesis. I have no major revisions to suggest.

Referee #2 (Comments on Novelty/Model System for Author):

experiments used are not solid. thus, no conclusions can be drawn that support the authors ´ hypothesis.

Referee #2 (Remarks for Author):

Unfortunately, the authors did not make a serious attempt to adress my major concerns. Particularly, regarding comment #1 and #3. Findings regarding comment#1 remain mostly in silico with no attempt to verify findings (at least some of them) experimentally. The most critical point however is comment#3. It is at this stage not possible to drwa meaningful conclusions from the animal experiment. A dose of pfu 5 is claimed to be lethal for 2009 pH1N1. This is very unusual. The authors should show LD50 data. Using only 4 animals per group is too low sample size not allowing robust conclusion. Humane endpoints are not defined, which are critical.

Point-to-point response

Review 2’s comment.

Unfortunately, the authors did not make a serious attempt to adress my major concerns. Particularly, regarding comment #1 and #3. Findings regarding comment#1 remain mostly in silico with no attempt to verify findings (at least some of them) experimentally. The most critical point however is comment#3. It is at this stage not possible to draw meaningful conclusions from the animal experiment. A dose of pfu 5 is claimed to be lethal for 2009 pH1N1. This is very unusual. The authors should show LD50 data. Using only 4 animals per group is too low sample size not allowing robust conclusion. Humane endpoints are not defined, which are critical.

A: We thank the reviewer’s comments. we are introducing the mouse experiment in more detail in the revised manuscript on page 17. In this mouse model, the LD50 of the mouse-adapted H1N1 virus is 150 pfu based on our previous study (Zheng, Chan et al., 2010). As we have mentioned in the previous response letter, 3-time intranasal inoculations of SPINK6 protein solutions befor e and after the virus inoculation substantially exacerbated the infection. It has been documented that a much lower pfu inoculation should be done if the inoculated mice are intranasally administrated for intervention (Smee, von Itzstein et al., 2012).

We agree with the reviewer on the issue of the sample size of the mouse experiment. We have repeated the mouse experiment with more mice and two time-points. The new data is presented in Figure 5C.

In the previous revision, we have specified the humane endpoint, which may have been missed by the reviewer. We highlighted it on page 17.

References:

Smee DF, von Itzstein M, Bhatt B, Tarbet EB (2012) Exacerbation of influenza virus infections in mice by intranasal treatments and implications for evaluation of antiviral drugs. *Antimicrob Agents Chemother* 56: 6328-33

Zheng B, Chan KH, Zhang AJ, Zhou J, Chan CC, Poon VK, Zhang K, Leung VH, Jin DY, Woo PC, Chan JF, To KK, Chen H, Yuen KY (2010) D225G mutation in hemagglutinin of pandemic influenza H1N1 (2009) virus enhances virulence in mice. *Exp Biol Med (Maywood)* 235: 981-8

26th Oct 2021

Dear Dr. Zhou,

Thank you for the submission of your manuscript to EMBO Molecular Medicine. I am pleased to inform you that we will be able to accept your manuscript pending the following final amendments:

1) In the main manuscript file, please do the following:
- Correct/answer the track changes suggested by our data editors by working from the attached document.
- Make sure that all special characters display well.
- In M&M, a statistical paragraph should reflect all information that you have filled in the Authors Checklist, especially regarding randomization, blinding, replication.
- In M&M, please include statement that the informed consent was obtained from all human subjects and that the experiments conformed to the principles set out in the WMA Declaration of Helsinki and the Department of Health and Human Services Belmont Report.
2) Synopsis:
- Synopsis text: Please submit the synopsis text as a separate .doc file.
- Synopsis image: Please provide the synopsis image as a 550 px-wide x (250-400)-px high high-resolution jpeg file.
- Please check your synopsis text and image, revise them if necessary and submit their final versions with your revised manuscript. Please be aware that in the proof stage minor corrections only are allowed (e.g., typos).
3) For more information: There is space at the end of each article to list relevant web links for further consultation by our readers. Could you identify some relevant ones and provide such information as well? Some examples are patient associations, relevant databases, OMIM/proteins/genes links, author's websites, etc...
4) As part of the EMBO Publications transparent editorial process initiative (see our Editorial at http://embomolmed.embopress.org/content/2/9/329), EMBO Molecular Medicine will publish online a Review Process File (RPF) to accompany accepted manuscripts. This file will be published in conjunction with your paper and will include the anonymous referee reports, your point-by-point response and all pertinent correspondence relating to the manuscript. Let us know whether you agree with the publication of the RPF and as here, if you want to remove or not any figures from it prior to publication. Please note that the Authors checklist will be published at the end of the RPF.
5) Please provide a point-by-point letter INCLUDING my comments as well as the reviewer's reports and your detailed responses (as Word file).

I look forward to reading a new revised version of your manuscript as soon as possible.

Yours sincerely,

Zeljko Durdevic

Zeljko Durdevic
Editor
EMBO Molecular Medicine

***** Reviewer's comments *****

Referee #2 (Remarks for Author):

The additional animal experiments performed have now strengthened the conclusions drawn. Figure 5 is now convincing.

The authors performed the requested editorial changes.

We are pleased to inform you that your manuscript is accepted for publication and is now being sent to our publisher to be included in the next available issue of EMBO Molecular Medicine.

EMBO PRESS

YOU MUST COMPLETE ALL CELLS WITH A PINK BACKGROUND ↓

PLEASE NOTE THAT THIS CHECKLIST WILL BE PUBLISHED ALONGSIDE YOUR PAPER

Corresponding Author Name: Jie Zhou

Journal Submitted to: EMBO Molecular Medicine
Manuscript Number: EMM-2021-14485

USEFUL LINKS FOR COMPLETING THIS FORM

http://www.antibodypedia.com
http://1degreebio.org
http://www.equator-network.org/reporting-guidelines/improving-bioscience-research-repor
http://grants.nih.gov/grants/olaw/olaw.htm
http://www.mrc.ac.uk/Ourresearch/Ethicsresearchguidance/Useofanimals/index.htm
http://ClinicalTrials.gov
http://www.consort-statement.org
http://www.consort-statement.org/checklists/view/32-consort/66-title
http://www.equator-network.org/reporting-guidelines/reporting-recommendations-for-tumo
http://datadryad.org
http://figshare.com
http://www.ncbi.nlm.nih.gov/gap
http://www.ebi.ac.uk/ega
http://biomodels.net/
http://biomodels.net/miriam/
http://jjj.biochem.sun.ac.za
https://osp.od.nih.gov/biosafety-biosecurity-and-emerging-biotechnology/
http://www.selectagents.gov/

Reporting Checklist For Life Sciences Articles (Rev. June 2017)

This checklist is used to ensure good reporting standards and to improve the reproducibility of published results. These guidelines are consistent with the Principles and Guidelines for Reporting Preclinical Research issued by the NIH in 2014. Please follow the journal's authorship guidelines in preparing your manuscript.

A- Figures
1. Data
The data shown in figures should satisfy the following conditions:

- the data were obtained and processed according to the field's best practice and are presented to reflect the results of the experiments in an accurate and unbiased manner.
- figure panels include only data points, measurements or observations that can be compared to each other in a scientifically meaningful way.
- graphs include clearly labeled error bars for independent experiments and sample sizes. Unless justified, error bars should not be shown for technical replicates.
- if n< 5, the individual data points from each experiment should be plotted and any statistical test employed should be justified
- Source Data should be included to report the data underlying graphs. Please follow the guidelines set out in the author ship guidelines on Data Presentation.

2. Captions

Each figure caption should contain the following information, for each panel where they are relevant:

- a specification of the experimental system investigated (eg cell line, species name).
- the assay(s) and method(s) used to carry out the reported observations and measurements
- an explicit mention of the biological and chemical entity(ies) that are being measured.
- an explicit mention of the biological and chemical entity(ies) that are altered/varied/perturbed in a controlled manner.
- the exact sample size (n) for each experimental group/condition, given as a number, not a range;
- a description of the sample collection allowing the reader to understand whether the samples represent technical or biological replicates (including how many animals, litters, cultures, etc.).
- a statement of how many times the experiment shown was independently replicated in the laboratory.
- definitions of statistical methods and measures:
  - common tests, such as t-test (please specify whether paired vs. unpaired), simple χ2 tests, Wilcoxon and Mann-Whitney tests, can be unambiguously identified by name only, but more complex techniques should be described in the methods section;
  - are tests one-sided or two-sided?
  - are there adjustments for multiple comparisons?
  - exact statistical test results, e.g., P values = x but not P values < x;
  - definition of 'center values' as median or average;
  - definition of error bars as s.d. or s.e.m.

Any descriptions too long for the figure legend should be included in the methods section and/or with the source data.

In the pink boxes below, please ensure that the answers to the following questions are reported in the manuscript itself. Every question should be answered. If the question is not relevant to your research, please write NA (non applicable). We encourage you to include a specific subsection in the methods section for statistics, reagents, animal models and human subjects.

B- Statistics and general methods

Please fill out these boxes ↓ (Do not worry if you cannot see all your text once you press return)

1.a. How was the sample size chosen to ensure adequate power to detect a pre-specified effect size?	Based on common practice.
1.b. For animal studies, include a statement about sample size estimate even if no statistical methods were used.	Yes.
2. Describe inclusion/exclusion criteria if samples or animals were excluded from the analysis. Were the criteria pre-established?	No sample was exlucded from analysis.
3. Were any steps taken to minimize the effects of subjective bias when allocating animals/samples to treatment (e.g. randomization procedure)? If yes, please describe.	Yes.
For animal studies, include a statement about randomization even if no randomization was used.	Yes.
4.a. Were any steps taken to minimize the effects of subjective bias during group allocation or/and when assessing results (e.g. blinding of the investigator)? If yes please describe.	Yes.
4.b. For animal studies, include a statement about blinding even if no blinding was done	Yes.
5. For every figure, are statistical tests justified as appropriate?	Yes.
Do the data meet the assumptions of the tests (e.g., normal distribution)? Describe any methods used to assess it.	Yes.
Is there an estimate of variation within each group of data?	No.

Is the variance similar between the groups that are being statistically compared?	No.

C- Reagents

6. To show that antibodies were profiled for use in the system under study (assay and species), provide a citation, catalog number and/or clone number, supplementary information or reference to an antibody validation profile. e.g., Antibodypedia (see link list at top right), 1DegreeBio (see link list at top right).	α-H1 (Sino Biological, 11055-T62); α-H7 (Sino Biological, 40103-RP02); α-SPINK6 (Abcam, ab110830); α-nucleoprotein of IAV (Novus, NBP2-16965); α-SPINK6 (Abnova, H00404203-M04); α-HAT (Thermo Fisher Scientific, PA5-42876)
7. Identify the source of cell lines and report if they were recently authenticated (e.g., by STR profiling) and tested for mycoplasma contamination.	BHK21, 293T and A549 cells are purchased from ATCC.

* for all hyperlinks, please see the table at the top right of the document

D- Animal Models

8. Report species, strain, gender, age of animals and genetic modification status where applicable. Please detail housing and husbandry conditions and the source of animals.	Female Balb/c mice of 6-8 weeks old were maintained in standard Biosafety level 2 animal laboratory and given access to standard pellet feed and water ad libitum.
9. For experiments involving live vertebrates, include a statement of compliance with ethical regulations and identify the committee(s) approving the experiments.	In all animal experiments, we followed the operating procedures approved by the Committee on the Use of Live Animals in Teaching and Research, the University of Hong Kong.
10. We recommend consulting the ARRIVE guidelines (see link list at top right) (PLoS Biol. 8(6), e1000412, 2010) to ensure that other relevant aspects of animal studies are adequately reported. See author guidelines, under 'Reporting Guidelines'. See also: NIH (see link list at top right) and MRC (see link list at top right) recommendations. Please confirm compliance.	Confirm compliance.

E- Human Subjects

11. Identify the committee(s) approving the study protocol.	NA
12. Include a statement confirming that informed consent was obtained from all subjects and that the experiments conformed to the principles set out in the WMA Declaration of Helsinki and the Department of Health and Human Services Belmont Report.	NA
13. For publication of patient photos, include a statement confirming that consent to publish was obtained.	NA
14. Report any restrictions on the availability (and/or on the use) of human data or samples.	NA
15. Report the clinical trial registration number (at ClinicalTrials.gov or equivalent), where applicable.	NA
16. For phase II and III randomized controlled trials, please refer to the CONSORT flow diagram (see link list at top right) and submit the CONSORT checklist (see link list at top right) with your submission. See author guidelines, under 'Reporting Guidelines'. Please confirm you have submitted this list.	NA
17. For tumor marker prognostic studies, we recommend that you follow the REMARK reporting guidelines (see link list at top right). See author guidelines, under 'Reporting Guidelines'. Please confirm you have followed these guidelines.	NA

F- Data Accessibility

18: Provide a "Data Availability" section at the end of the Materials & Methods, listing the accession codes for data generated in this study and deposited in a public database (e.g. RNA-Seq data: Gene Expression Omnibus GSE39462, Proteomics data: PRIDE PXD000208 etc.) Please refer to our author guidelines for 'Data Deposition'.<br><br>Data deposition in a public repository is mandatory for:<br>a. Protein, DNA and RNA sequences<br>b. Macromolecular structures<br>c. Crystallographic data for small molecules<br>d. Functional genomics data<br>e. Proteomics and molecular interactions	NA
19. Deposition is strongly recommended for any datasets that are central and integral to the study; please consider the journal's data policy. If no structured public repository exists for a given data type, we encourage the provision of datasets in the manuscript as a Supplementary Document (see author guidelines under 'Expanded View' or in unstructured repositories such as Dryad (see link list at top right) or Figshare (see link list at top right).	NA
20. Access to human clinical and genomic datasets should be provided with as few restrictions as possible while respecting ethical obligations to the patients and relevant medical and legal issues. If practically possible and compatible with the individual consent agreement used in the study, such data should be deposited in one of the major public access-controlled repositories such as dbGAP (see link list at top right) or EGA (see link list at top right).	The Genotype-Tissue Expression (GTEx) Project was supported by the Common Fund of National Institutes of Health, and by NCI, NHGRI, NHLBI, NIDA, NIMH, and NINDS. The data used for the analyses described in this manuscript were obtained from GTEx Portal, GTEx Analysis Release V8 (dbGaP Accession phs000424.v8.p2) on 12/01/2020.
21. Computational models that are central and integral to a study should be shared without restrictions and provided in a machine-readable form. The relevant accession numbers or links should be provided. When possible, standardized format (SBML, CellML) should be used instead of scripts (e.g. MATLAB). Authors are strongly encouraged to follow the MIRIAM guidelines (see link list at top right) and deposit their model in a public database such as Biomodels (see link list at top right) or JWS Online (see link list at top right). If computer source code is provided with the paper, it should be deposited in a public repository or included in supplementary information.	NA

G- Dual use research of concern

22. Could your study fall under dual use research restrictions? Please check biosecurity documents (see link list at top right) and list of select agents and toxins (APHIS/CDC) (see link list at top right). According to our biosecurity guidelines, provide a statement only if it could.	NA
